# Honeysuckle-derived nanovesicles alleviate inflammatory bowel disease-associated neuropsychiatric disorders via the gut–brain axis

**DOI:** 10.7150/thno.132241

**Published:** 2026-06-10

**Authors:** Ailin Wu, Qin Chu, Le Ding, Yan Zeng, Liqiang Shao, Yuanyuan Wang, Yuanhao Zhou, Weijiao Fan, Xin Zhang, Jianwei Wang, Jie Wang, Shunyuan Guo, Ketao Jin, Xiaoru Chang, Xiaozhou Mou

**Affiliations:** 1 Center for Rehabilitation Medicine, Rehabilitation & Sports Medicine Research Institute of Zhejiang Province, Department of Rehabilitation Medicine, Zhejiang Provincial People's Hospital, Affiliated People's Hospital, Hangzhou Medical College, Hangzhou, Zhejiang, China.; 2 Department of Neurology, Center for Rehabilitation Medicine, Zhejiang Provincial People's Hospital, Affiliated People's Hospital, Hangzhou Medical College, Hangzhou, Zhejiang, China.; 3 College of Pharmacy, Zhejiang University of Technology, Hangzhou, Zhejiang, China.; 4 EVitalBio (Hangzhou) Co., Ltd, Hangzhou, Zhejiang, China.; 5 Department of Colorectal and Anal Surgery, The First Affiliated Hospital of Zhejiang Chinese Medical University. Hangzhou, Zhejiang 310003, China.

**Keywords:** inflammatory bowel disease, anxiety and depression, honeysuckle-derived nanovesicles, gut–brain axis, tryptophan metabolism

## Abstract

**Rationale:**

Inflammatory bowel disease (IBD) is frequently accompanied by anxiety and depression, yet effective therapies targeting both intestinal inflammation and neuropsychiatric symptoms remain limited. This study aimed to investigate whether honeysuckle-derived nanovesicles (HNVs) could alleviate IBD-related affective disturbances by modulating the microbial-gut-brain axis (MGBA).

**Methods:**

A chronic colitis mouse model was established using dextran sulfate sodium (DSS). Mice were treated orally with HNVs, and anxiety- and depression-like behaviors were evaluated using behavioral assays. Intestinal barrier integrity, gut microbiota composition, systemic immune responses, proinflammatory cytokine production, hippocampal immune infiltration, 5-hydroxytryptamine (5-HT) biosynthesis and metabolism, and activation of the BDNF/TrkB/GSK3β signaling pathway were assessed using histological, microbiological, immunological, biochemical, and molecular analyses.

**Results:**

Oral administration of HNVs markedly attenuated anxiety- and depression-like behaviors in mice with chronic colitis. HNV treatment preserved intestinal barrier integrity, reshaped the gut microbial community, modulated systemic immune responses, reduced proinflammatory cytokine production and hippocampal inflammatory infiltration, and regulated 5-HT biosynthesis and metabolic pathways. These effects were associated with restoration of intestinal and neural homeostasis and activation of the BDNF/TrkB/GSK3β signaling cascade.

**Conclusions:**

HNVs alleviated IBD-related affective disturbances in experimental chronic colitis by regulating the MGBA, suppressing inflammation, modulating 5-HT metabolism, and restoring intestinal and neural homeostasis. These findings support HNVs as a potential natural nanotherapeutic strategy for the integrated management of intestinal inflammation and associated neuropsychiatric comorbidities.

## Introduction

Inflammatory bowel disease (IBD) refers to a group of intricate conditions marked by persistent inflammation within the gastrointestinal tract. The two primary forms of IBD are Crohn's disease (CD) and ulcerative colitis (UC) 1. The incidence of IBD has been increasing at a consistent rate in recent years, placing significant pressure on patients’ quality of life and mental well-being 2, 3. Research findings suggest that over half of the IBD population exhibits symptoms of anxiety and depression, a phenomenon referred to as gut-brain comorbidity, which has become a prominent issue in current clinical management 2. However, the application of traditional antidepressant or anti-anxiety drugs in IBD patients is often subject to problems such as limited efficacy, large side effects, and conflicts with primary disease treatment. There is an immediate demand for the creation of novel intervention approaches that possess both anti-inflammatory and mental regulatory properties.

The gut-brain axis (GBA), a bidirectional signaling pathway between the gastrointestinal tract and the central nervous system (CNS), plays a critical role in the development of IBD and related neuropsychiatric disorders 4, 5. Chronic intestinal inflammation and neuroinflammation may be driven by alterations in gut microbiota composition and their metabolic activity through the microbiota-gut-brain axis (MGBA). After the intestinal barrier is destroyed, pro-inflammatory microorganisms such as lipopolysaccharides (LPS) and inflammatory factors will enter the bloodstream, leading to systemic inflammatory reactions 6. Circulating inflammatory mediators may translocate across the blood-brain barrier (BBB) into key brain regions such as the hippocampus, where they promote neuroinflammation and neuronal damage, thereby contributing to neuropsychiatric disorders. Serotonin (5-hydroxytryptamine, 5-HT) is an important mediator of the gut-brain axis (GBA). Approximately 90-95% of 5-HT is produced in the intestine, where it regulates intestinal motility and inflammatory responses, and may influence mood through the gut–brain axis 7, 8. The study demonstrated that alterations in 5-HT synthesis, metabolism, and receptor expression in patients with IBD impair CNS function and exacerbate anxiety- and depression-like behaviors 9. Therefore, the targeted 5-HT signal pathway can be regulated or dual intervention for IBD and its related mental disorders.

Plant-derived exosome-like nanovesicles (PELNVs) have broad application prospects in intestinal diseases due to their good biocompatibility, gastrointestinal stability, and cross-barrier delivery ability, enabling them to remain stable in the gastrointestinal environment, target the colon, and be ingested by intestinal symbiotic bacteria 6, 10, 11. By regulating intestinal immune and inflammatory responses, they can alleviate the development of IBD 12. Honeysuckle, a traditional Chinese medicine (TCM), is famous for its heat-clearing effect, detoxifying, anti-inflammatory and antibacterial 13, 14. The honeysuckle-derived nanovesicles (HNVs) extracted from it show the potential of safe and efficient treatment through the multi-dimensional synergy of anti-inflammatory, barrier repair, immune regulation and flora metabolic regulation. It provides new ideas for the development of naturally derived vesicles to alleviate IBD treatment strategies 15. In recent years, increasing evidence has indicated that PELNVs possess therapeutic potential for IBD. For instance, turmeric-derived exosome-like nanovesicles (TDNPs) accumulate at sites of colonic inflammation, where they exert anti-inflammatory effects, promote mucosal repair, and enhance intestinal barrier function in experimental colitis models 16. Similarly, Garlic-derived exosome-like nanovesicles (GENs) have been reported to alleviate DSS-induced colitis, suggesting that PELNVs possess natural suitability for oral delivery and potential for intestinal immune modulation 17. Furthermore, the application potential of PELNVs in brain diseases and neuroinflammation is rapidly gaining attention. Their advantages include a broad source, low toxicity, low immunogenicity, and plasticity as natural nanodelivery platforms 18. However, compared to the considerable animal experimental support in the IBD field, direct therapeutic evidence for their use in neuropsychiatric disorders remains relatively limited. In this context, the innovation of HNVs lies not only in the general characteristics of PELNVs, but also in their focus on the GBA, which connects the control of intestinal inflammation with the improvement of neuropsychiatric outcomes, thereby offering a more integrated intervention strategy for IBD-related neuropsychiatric comorbidities.

Based on the concept of the same origin of medicinal food, this study systematically explores the molecular mechanism of HNVs that affect the GBA function by regulating the intestinal 5-HT metabolism, thereby alleviating IBD-related anxiety and depression. Based on the perspective of MGBA, the mechanism of HNVs intervention in 5-HT metabolism to alleviate IBD comorbidity is deeply revealed, which is of great significance to the construction of a new comorbidity intervention system and its clinical transformation. We studied the therapeutic effect of oral HNVs on IBD and related anxiety and depression. Oral HNVs corrected DSS-induced gut microbiota dysbiosis and attenuated systemic LPS and pro-inflammatory cytokine levels associated with intestinal barrier dysfunction, potentially through modulation of microbiota-driven 5-HT metabolism. HNVs regulate tryptophan (Trp) metabolism by modulating the gut microbiota, promoting the expansion of beneficial bacteria while suppressing pathogenic taxa. This is associated with upregulation of tryptophan hydroxylase 1 (TPH1) and downregulation of indoleamine 2,3-dioxygenase 1 (IDO1), thereby shifting Trp metabolism from the kynurenine (Kyn) pathway toward 5-HT synthesis. By upregulating Tryptophan hydroxylase 2 (TPH2) and 5-Hydroxytryptophan (5-HTP) in the brain, the 5-HT content in brain tissue is increased, and then improving IBD-related anxiety and depressive comorbidity symptoms through the BDNF/TrkB/GSK3β pathway. This study is expected to expand the new strategy for the treatment of IBD-related mental disorders with microbial regulation as the core, and promote the modernization and transformation of TCM resources.

## Results

### Preparation and characterization of HNVs

**Figure 1:**
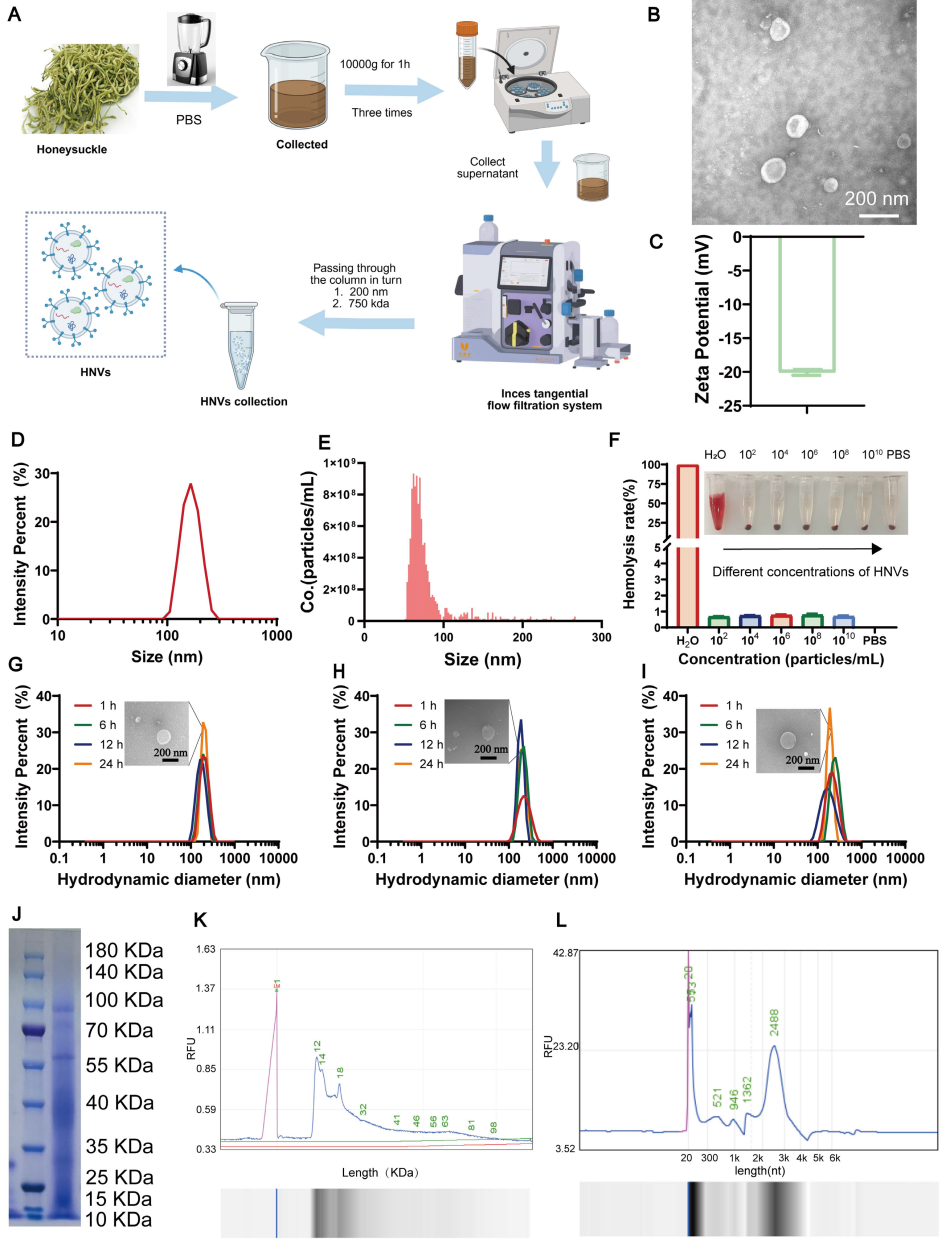
**Preparation flow chart of HNVs and its characterization.** (A) Schematic illustration of the HNV extraction procedure. (B) TEM characterization of the morphology and size of HNVs. (C) Zeta potential of HNVs. (D) DLS analysis of the size distribution of HNVs. (E) NanoCoulter counter analysis of the vesicle count of HNVs. (F) Hemolysis assay of HNVs (n = 3). (G-I) Particle size distribution of HNVs after incubation at 37°C in PBS (G), simulated gastric fluid (H), and simulated intestinal fluid (I). (J) Coomassie brilliant blue staining. (K-L) HNVs proteins and RNAs were separated and analyzed based on molecular weight using a bio-fragment analyzer.

HNVs are separated by ultra-fast centrifugal combined with tangent flow filtration technology (Figure 1A). Transmission electron microscopy (TEM) shows that HNVs have a typical round or cup-shaped shape (Figure 1B). An average zeta potential of -18 mV and a mean particle size of approximately 180 nm were determined for HNVs by DLS analysis (Figure 1C-D). The vesicle concentration of HNVs, measured using a NanoCoulter counter, was 2.21 × 10¹² particles/mL (Figure 1E). The results of mouse hemolysis experiments show that the hemolysis rate of HNVs is low, and all concentrations are within 1%, indicating that HNVs have good biocompatibility (Figure 1F). In the gastrointestinal simulation solution and PBS, the particle size of HNVs can be maintained stable for at least 24h. TEM analysis revealed that the particle size remained largely unchanged in simulated gastrointestinal fluids, indicating that HNVs have good stability in gastric juice and intestinal fluid (Figure 1G-I). Subsequently, the HNVs were subjected to membrane disruption treatment. TEM and NanoCoulter counter analysis confirmed the destruction of the HNV membrane, and no vesicular structures were observed in the honeysuckle decoction (HD) fraction (Figure S1A-C). Polyacrylamide gel electrophoresis (PAGE) showed protein bands between 10 and 100 KDa (Figure 1J), and lipid profiling confirmed the presence of various lipid types (Figure 1K). RNA analysis further revealed that the majority of RNA contained in HNVs consisted of small RNA species (Figure 1L). We performed untargeted metabolomic analysis of HNVs and found that lipids were the most abundant components, followed by organic compounds (Figure S1D). Within the lipid category, flavonoids were the most abundant (Figure S1E). Among compounds with biological roles, carboxylic acids and amino acids were predominant (Figure S1F). KEGG pathway enrichment analysis demonstrated that HNVs were enriched in pathways associated with tyrosine metabolism, flavonoid and flavonol biosynthesis, and tryptophan metabolism (Figure S1G). These compositional features provide mechanistic insight into the role of HNVs in modulating the gut microbiota to alleviate IBD and associated neuropsychiatric disorders. Collectively, these findings highlight the favorable biocompatibility and structural stability of HNVs. More importantly, they imply that HNVs are capable of preserving their functional integrity following oral administration, a property that could be crucial for their subsequent biological applications.

### Relief of colitis related mental disorders

**Figure 2:**
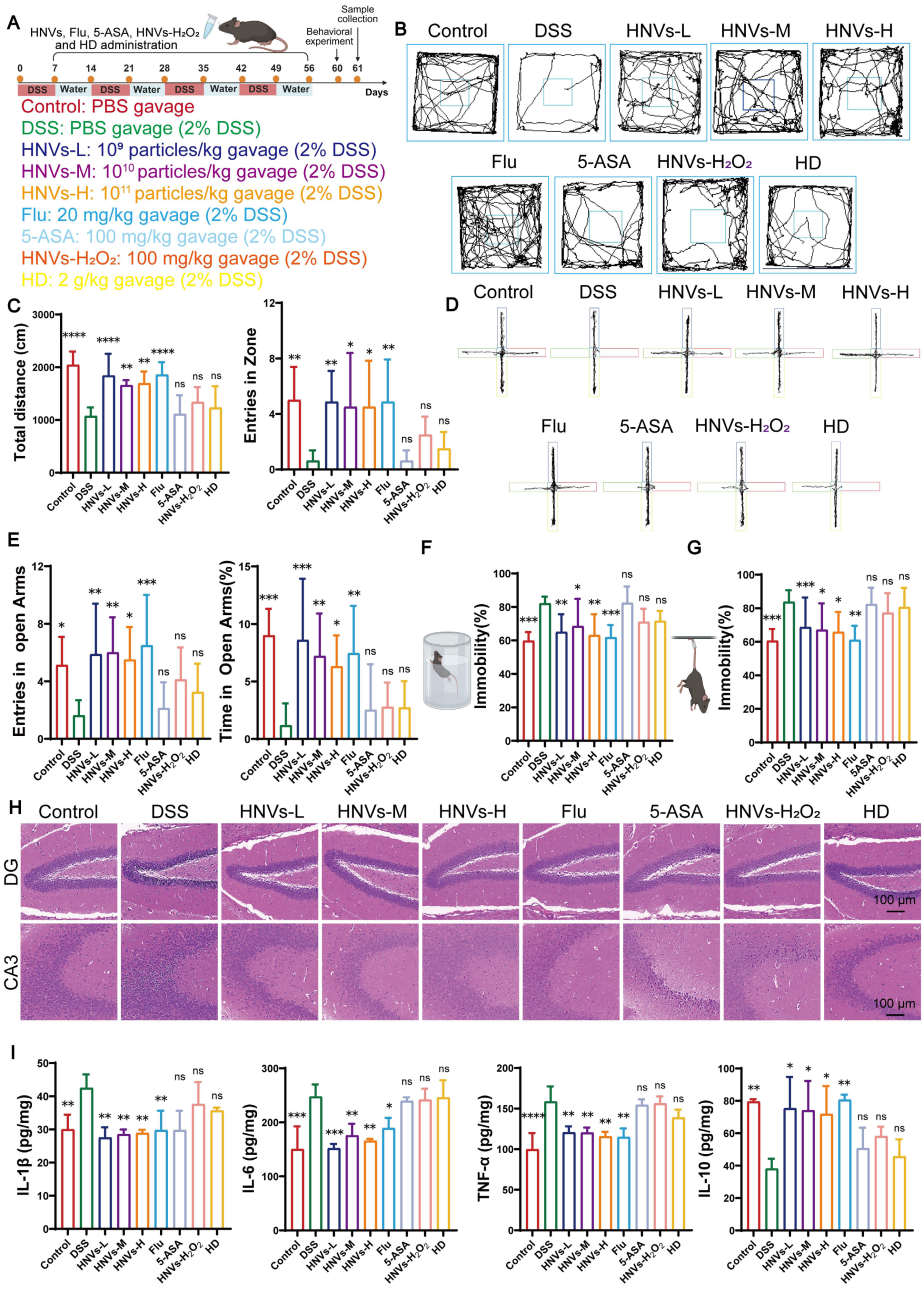
**HNVs alleviate colitis-associated neuropsychiatric disorders by regulating neurons.** (A) A schematic diagram illustrating the DSS-induced chronic colitis model in mice. (B) Mouse movement tracks in the OFT. (C) The total distance of mice in OFT and the number of times they enter the central region (n = 8). (D) Representative mouse movement tracks in the EPM. (E) The number of times mice entered the EPM arm opening and the percentage of staying in the opening arm (n = 8). (F) Duration of immobility observed in mice during the FST (n = 8). (G) Time spent immobile by mice in the TST assay (n = 8). (H) Representative H&E-stained brain sections from each group. Scale bar = 100 μm. (I) ELISA analysis of inflammatory cytokine expression in brain from different mouse groups (n = 3). All data are expressed as mean ± SD. Statistical annotations: ns, not significant (*p* ≥ 0.05); *p < 0.05; **p < 0.01; ***p < 0.001; ****p < 0.0001.

Previous studies have reported the treatment of UC with exosome-like nanocapsules at doses ranging from 10^9^ to 10^11^ particles/kg 19-21. Based on these findings, HNVs were evaluated at low (10⁹ particles/kg), medium (10¹⁰ particles/kg), and high (10¹¹ particles/kg) doses, with fluoxetine (Flu, 10 mg/kg) 22, 23 and 5-ASA (100 mg/kg) used as positive controls 11. To elucidate the progression of anxiety and depression like manifestations during IBD and to evaluate long-term treatment effects of HNVs on both intestinal pathology and related behavioral symptoms, we constructed a chronic IBD mouse model. DSS was used to induce inflammation resembling UC. To induce chronic colitis, mice were subjected to four cycles of DSS administration, each consisting of 1 week of DSS and followed by 1 week of recovery (Figure 2A) 24. Starting from the first recovery period after the initial DSS cycle and continuing until the end of the fourth recovery period, mice in the treatment groups were orally gavaged daily with 200 µL of HNVs-L (10⁹ particles/mL), HNVs-M (10¹⁰ particles/mL), HNVs-H (10^11^ particles/mL), Flu (10 mg/kg), 5-ASA (100 mg/kg), HNVs+H_2_O_2_ (100 mg/kg) and HD (2 g/kg). Mice in the DSS and control groups received equal volumes of PBS, whereas control mice were maintained on normal drinking water without DSS throughout the experiment. Behavioral experiments were conducted after the fourth recovery phase to avoid the influence of pathological behaviors induced by physical illness or pain in the mice. In the open field test (OFT), mice in the DSS, 5-ASA, HNVs+H_2_O_2_, and HD groups exhibited reduced exploratory behavior, as evidenced by decreased locomotor activity and limited central area exploration. By contrast, mice treated with HNVs and Flu exhibited more diverse activity and irregular movement patterns. Additionally, the total distance traveled by colitic mice was also significantly increased after treatment with HNVs or Flu (Figure 2B-C), indicating a decrease in anxiety-like behavior. Similarly, in the elevated plus maze (EPM), mice in the DSS, 5-ASA, HNVs+H_2_O_2_, and HD groups showed reduced exploration of the open arms, spending less time there and entering the open arms fewer times (Figure 2D-E). These results suggest that HNV and Flu treatments alleviate anxiety- and depression-like behaviors in mice. Behavioral despair was further assessed using the tail suspension test (TST) and forced swim test (FST). DSS, 5-ASA, HNVs+H_2_O_2_, and HD groups displayed significantly increased immobility durations compared with the HNVs, Flu and control groups (Figure 2F-G), corroborating that HNVs treatment was associated with reduced despair like behavior. Taken together, these results show that oral HNVs treatment alleviates colitis-associated neuropsychiatric disturbances across multiple endpoints.

### HNVs alleviate mental disorders by modulating neurons

Drawing from the behavioral experimental outcomes, we propose that mental disorders associated with colitis could be connected to CNS damage. Histological evaluation of brain tissues indicated that DSS exposure led to pronounced lesions in the dentate gyrus as well as the hippocampus CA3 region, whereas the extent of injury was notably alleviated in the HNVs-treated group. Although the 5-ASA, HNVs + H_2_O_2_, and HD groups also showed therapeutic benefits relative to the DSS group, their efficacy was inferior to that of the HNVs and Flu groups (Figure 2H). Nissl staining is widely used to evaluate neural tissue structure and injury. Application of this method revealed that the DSS group exhibited fewer viable neurons within the hippocampus dentate gyrus and CA3 subfields. However, HNVs and Flu treatment successfully reversed this decrease, and the effect is more significant than that of the 5-ASA, HNVs + H_2_O_2_, and HD group (Figure S2A).

Chronic intestinal inflammation in IBD disrupts intestinal epithelial barrier function, promoting bacterial translocation and elevating circulating inflammatory mediators 25, 26. This persistent gut derived systemic inflammation can access the CNS via pathways such as the BBB, thereby activating resident immune cells, including microglia 27, 28. Subsequently, the release of cytokines and chemokines can cause neuroinflammation, leading to neuronal dysfunction, and producing behavioral manifestations similar to anxiety and depression 29. Accordingly, inflammatory cytokine levels in mouse brain tissue were quantified by Enzyme-linked immunosorbent assay (ELISA). As shown in Figure 2I, the DSS, 5-ASA, HNVs + H₂O₂, and HD groups showed elevated pro-inflammatory cytokines (IL-1β, TNF-α, and IL-6) and reduced IL-10 levels relative to the HNV-treated and control groups. Relative to the HNV-treated group, the Flu group displayed elevated IL-10 levels, suggesting comparatively weaker anti-inflammatory efficacy. These results indicate that DSS-induced increases in pro-inflammatory cytokines may impair BBB integrity, facilitate the infiltration of inflammatory mediators into the brain, and thereby induce hippocampal inflammation. The level of 5-HT in the brain tissue of each group of mice was detected by ELISA to evaluate the neurotransmitter changes associated with anxiety and depression-like behavior. A marked decline in brain 5-HT concentrations was detected in DSS-treated mice relative to the control cohort, indicating possible disturbances in affective behavior. Notably, treatment with HNVs or Flu markedly restored brain 5-HT levels, whereas no significant improvement was observed in the 5-ASA, HNVs-H₂O₂, or HD groups (Figure S2B). Based on the results of the inflammation factor ELISA and 5-HT assays, HNVs-L showed superior efficacy compared to HNVs-M, HNVs-H, as well as the positive controls, Flu and 5-ASA. Additionally, the effects of HD and HNVs-H₂O₂ were inferior to those of HNVs. Therefore, HNVs-L was designated for subsequent experimentation and assigned the abbreviated name HNVs from this point forward.

During the progression of anxiety and depression, inflammation within the CNS may result from increased levels of pathogens, including LPS, proinflammatory cytokines, and bacteria circulating in blood 30, 31. Plasma LPS concentrations were quantified by ELISA. The DSS group showed markedly higher LPS levels compared to both the control and HNV-treated groups (Figure S2C). High LPS levels could be an important contributor to inflammation in the CNS 32. S100β calcium-binding proteinβ (S100β) is a marker of BBB damage. A pronounced elevation in plasma S100β concentrations was detected in the DSS group, implying compromised BBB integrity; notably, HNVs administration substantially attenuated this disruption. As resident immune cells of the CNS, microglia play a key role in the regulation of neuroinflammatory reactions (Figure S2D). Under conditions of BBB disruption, peripheral inflammatory factors can infiltrate the CNS and interact with receptors on the surface of microglia, thereby triggering their activation and exacerbating neuroinflammation 33. In the DSS-treated group, microglia in the mouse brain were markedly activated and exhibited a characteristic amoeboid morphology. However, HNVs treatment significantly reversed these changes (Figure S2E-F), indicating that it may play a role by reducing the generation and leakage of pathogenic factors and maintaining the integrity of the BBB. Moreover, HNVs may modulate neuroinflammatory responses by attenuating microglial activation. Systemic inflammation may arise from intestinal inflammation and barrier dysfunction, which facilitate the translocation of pro-inflammatory mediators into the circulation.

### *In vitro* evaluation of the anti-inflammatory effects of HNVs

The *in vitro* anti-inflammatory capacity of HNVs was evaluated using the RAW264.7 macrophage cell line. LPS stimulation increased inflammatory mediators and oxidative stress, as shown by higher ROS levels. RT-qPCR results indicated that LPS substantially upregulated TNF-α, IL-6, and IL-1β mRNA expression (Figure S3A). HNVs significantly suppressed these increases, suggesting potential therapeutic effects for inflammatory disorders. LPS also induced ROS generation in RAW264.7 cells, indicated by green fluorescence (Figure S3B). Flow cytometry analysis further confirmed that ROS generation induced by LPS was reduced in the HNVs group (Figure S3C). These results indicate that HNVs possess strong anti-inflammatory properties.

### Distribution of HNVs *in vivo* and *in vitro* cell uptake

**Figure 3:**
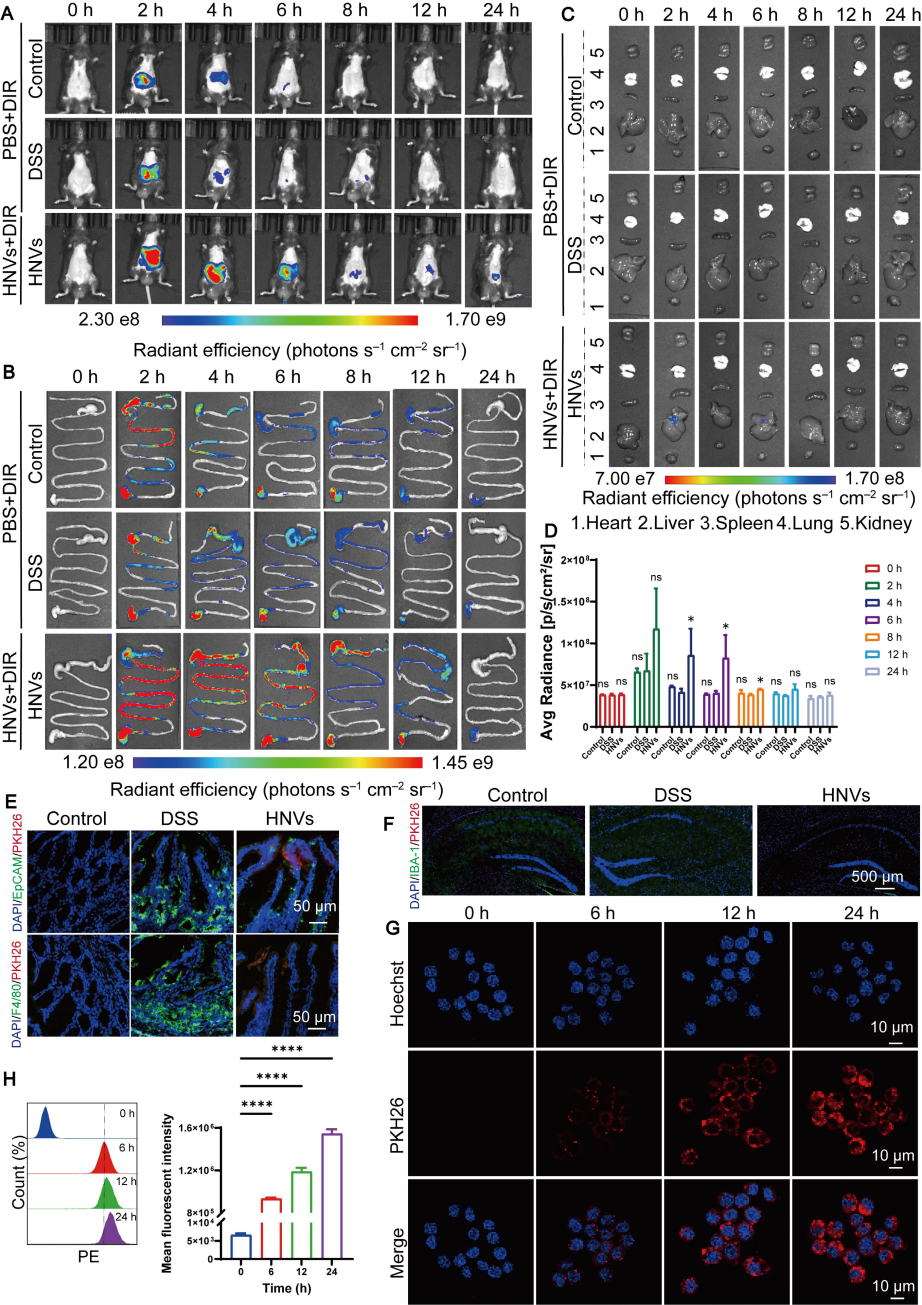
**Biological targeting and cell uptake of HNVs.** (A) Whole body imaging 0, 2, 4, 6, 8, 12, and 24 hours after oral HNVs in mice (B) Intestinal fluorescence of each group of mice. (C) Fluorescence distribution of major organs at different points in time. (D) *In vivo* fluorescence intensity analysis of mice (n = 3). (E) PKH26 co localization with intestinal epithelial cells and macrophages, Scale bar: 50 μm. (F) Co-localization of IBA and PKH26 in brain tissue, Scale bar: 500 μm. (G) Cell uptake at different points in time. Scale bar = 10 μm. (H) Flow cytometric analysis of the uptake and quantification of PKH26-labeled HNVs by RAW264.7 cells at different time points (n = 3). The data representation employs mean ± SD; statistical thresholds correspond to *p < 0.05, **p < 0.01, ***p < 0.001, and ****p < 0.0001.

In the previous experiments, we verified the relieving and *in vitro* anti-inflammatory effects of HNVs on IBD and its mental disorders. To investigate the mechanisms by which HNVs alleviate anxiety- and depression-like behaviors, their biodistribution was evaluated *in vivo* and *ex vivo*. HNVs (10^9^ particles/kg) co-labeled with DIR and PKH26 are used, and their fluorescence intensity is detected by imaging systems at 0, 2, 4, 6, 8, 12, and 24h. The fluorescence intensity of HNVs reached its highest point 2 hours after administration and subsequently declined over time (Figure 3A). Fluorescence intensity in the colon peaked at 2 h (Figure 3B), with HNVs also detected in the liver at this time point (Figure 3C). HNVs are mainly distributed in the colon. Fluorescence imaging performed *in vitro* revealed that at time points of 4, 6, and 8 hours, signal intensity in the HNVs-treated group markedly surpassed that observed in both the control and DSS cohorts (Figure 3D). HNV-treated mice exhibited significantly higher colonic fluorescence intensity than the DSS and control groups at 2, 6, 8, and 12 h, suggesting prolonged retention (Figure S4A) and improved local bioavailability. No significant intergroup differences were detected when mean fluorescence intensity was statistically evaluated in liver specimens (Figure S4B). HNVs were mainly distributed in the colon and liver, and their accumulation in the liver may be attributed to DSS-induced gut barrier damage; further co-staining of PKH26 with markers for intestinal macrophages, epithelial cells, and brain glial cells showed that HNVs were internalized by both intestinal macrophages and epithelial cells, but did not reach the brain (Figures 3E–F).

The uptake of substances by cells is a key factor influencing the effectiveness of drug delivery systems. To investigate how HNVs are internalized by RAW 264.7 macrophages, HNVs were tagged with the red fluorescent dye PKH26 and incubated with the cells. The results showed that macrophages actively took up HNVs, this was evidenced by a gradual accumulation of red fluorescence within the cells over time (Figure 3G-H). Collectively, these results support the possibility that HNVs could alleviate colitis-related mental disorders by modulating the GBA.

### HNVs can relieve chronic colitis induced by DSS

**Figure 4:**
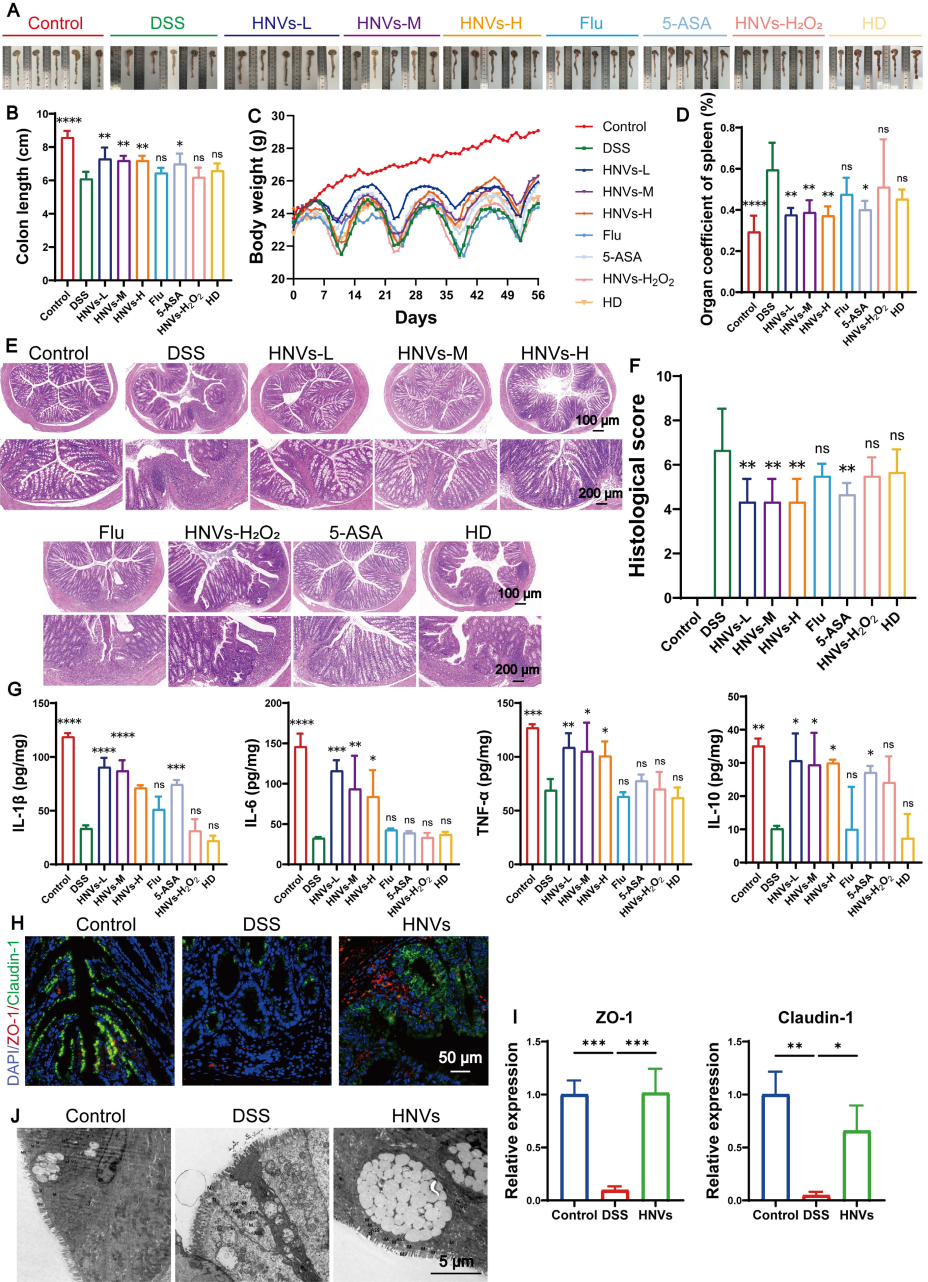
**HNVs alleviate DSS-induced intestinal inflammation.** (A) Anatomy of each group of colon tissue (n = 5). (B) Colon lengths of different groups (n = 5). (C) Body weight changes in each group (n = 5). (D) Spleen index across experimental groups (n = 6). (E) Colonic sections subjected to H&E staining. Magnification bars correspond to 100 μm and 200 μm for low- and high-power views, respectively. (F) Assessment of colon tissue histopathology using a standardized scoring system (n = 6). (G) Measurement of interleukin-1β, IL-6, TNF-α, and IL-10 protein abundance in colonic homogenates via ELISA (n = 3). (H) Immunofluorescence detection of tight junction markers ZO-1 (visualized in red) together with claudin-1 (green signal) across all experimental cohorts. The scale marker indicates 50 μm. (I) Quantification of ZO-1 and claudin-1 fluorescence intensities (n = 3). (J) Ultrastructural imaging of colon specimens through TEM. Scale indicator: 5 μm. All summarized data are displayed as the mean ± standard deviation; ns signifies statistically non-significant differences, whereas asterisks indicate *p < 0.05, **p < 0.01, ***p < 0.001, and ****p < 0.0001.

Based on *in vivo* biodistribution data, we evaluated the therapeutic efficacy of HNVs in a chronic colitis model. Oral administration of HNVs-L, HNVs-M and HNVs-H significantly improved symptoms induced by DSS, such as colon shortening (Figure 4A-B), weight loss (Figure 4C), splenomegaly (Figure S5A), abnormal spleen index ((Figure 4D), alleviated diarrhea, rectal bleeding (Figure S5B), and brain ischemia (Figure S5C). Histological analysis revealed that HNVs protected the intestinal epithelial structure and maintained crypt integrity (Figure 4E). The therapeutic effect of the 5-ASA group was inferior to that of the HNVs group, while the HNV-H₂O₂ and Flu groups exhibited lower efficacy than the 5-ASA group (Figure 4F). ELISA analysis of inflammatory cytokines in colon tissue revealed that the HNVs-L group exhibited significantly greater efficacy than the HNVs-M and HNVs-H groups, and its therapeutic effect surpassed that of the positive control drugs 5-ASA and Flu. In contrast, the HNVs-H₂O₂ and HD groups showed no appreciable therapeutic effect compared with the DSS group (Figure 4G). The HD group did not show obvious therapeutic benefits, probably because the HD formulation contained fewer exosome-like nanoparticles. According to the above experimental results, the HNVs treatment group in subsequent experiments will be administered at the HNVs-L concentration to ensure a reasonable and reproducible dosage. As demonstrated by immunohistochemistry, HNVs reduced the levels of IL-1β and other pro-inflammatory molecules in the intestinal mucosa, pointing to direct anti-inflammatory outcomes (Figure S5D).

### HNVs alleviate intestinal epithelial barrier dysfunction

The gut barrier constitutes a highly coordinated, multilayered system encompassing tight-junction-bound intestinal epithelia, a protective mucus stratum, immune effector cells positioned in gut-associated lymphoid structures, and the indigenous symbiotic microbiota 34. The disruption of tight junction protein expression and function results in a loss of intestinal barrier integrity 35. This leads to enhanced permeability of the intestinal mucosa, facilitating the passage of harmful substances and triggering inflammatory responses. Immunofluorescence staining of two intestinal adhesion proteins, after HNV treatment, Occludin-1 and ZO-1 staining revealed higher expression of both proteins, with more positive cells detected, indicating that HNVs treatment helps maintain the integrity of the colonic mucosal barrier (Figure 4 H-I).

Additionally, TEM images revealed moderate swelling of epithelial cells in the DSS group; tight junctions and desmosomes were blurred; mitochondria were severely swollen with matrix dissolution, cristae fragmentation, and localized vacuolation, and membrane structures were partially blurred. These pathological changes were alleviated by HNV administration, whereas the DSS group exhibited more severe epithelial injury than the HNV and control groups (Figure 4J). Overall, orally delivered HNVs reduce intestinal inflammatory responses and improve barrier integrity, this results in a reduction of DSS-induced colonic inflammation in a chronic colitis model.

### Effects of HNVs on gut microbial composition and metabolites

**Figure 5:**
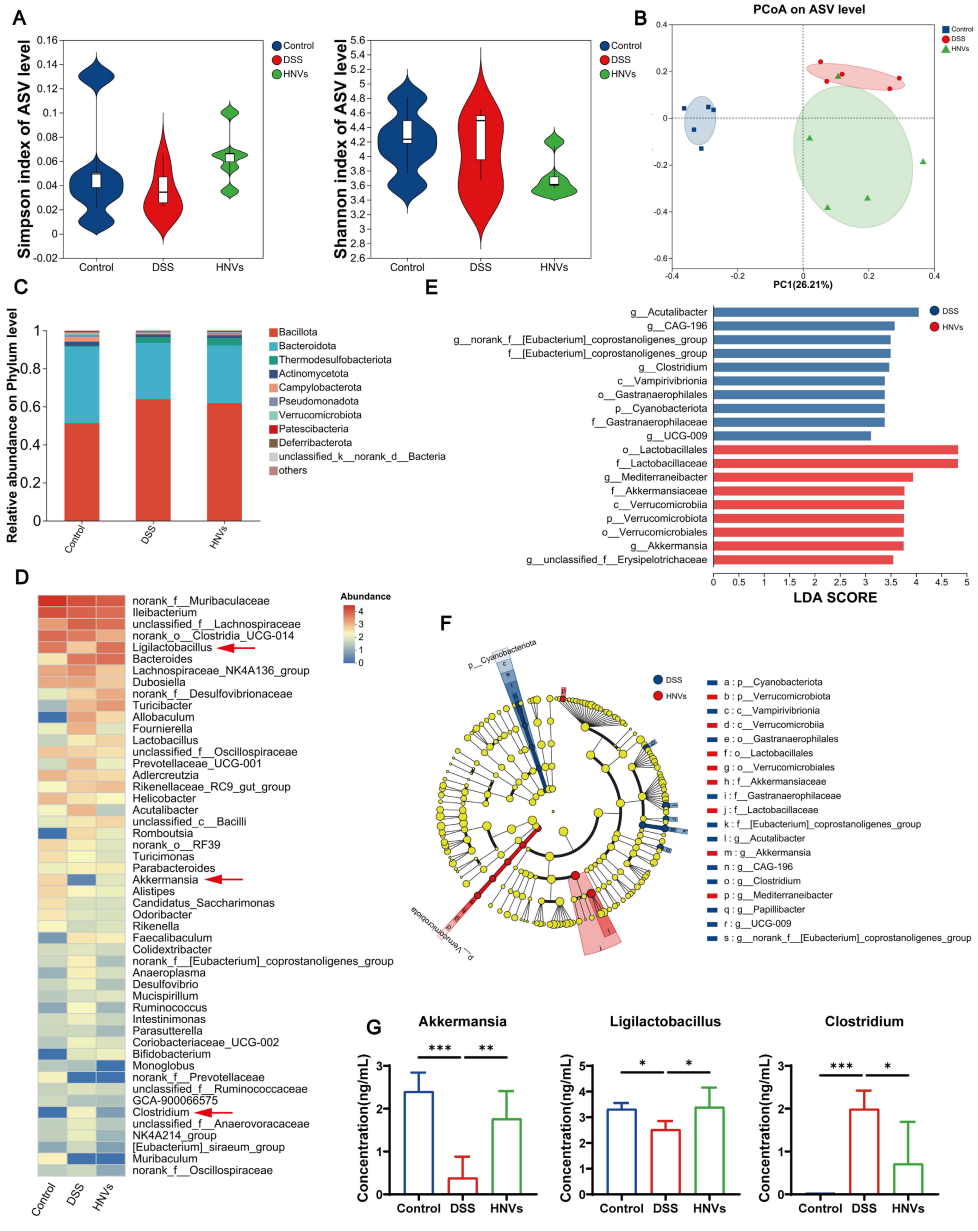
**Regulation of intestinal microbiota by HNVs.** (A) Comparison of α-diversity indices (Simpson and Shannon) across different groups. (B) PCoA plot (n = 5). (C) Phylum-level microbiota were analyzed using bar plots. (D) The relative abundance of genus level microbiota. (E-F) Taxonomic cladograms generated by LEfSe analysis. (G) Analysis of changes in bacterial genera (n = 5). Results are expressed as mean ± SD. Significance levels are denoted as *p < 0.05, **p < 0.01, and ***p < 0.001.

Gut microbiota alterations in the DSS-induced colitis model and their modulation by HNVs were characterized by 16S rRNA gene sequencing. Alpha diversity, as measured by the Simpson and Shannon indices, did not differ significantly among groups (Figure 5A), indicating that species richness remained relatively stable across different treatments. Beta diversity principal coordinate analysis (PCoA) demonstrated distinct separation of the DSS group from the control and HNV groups, reflecting significant changes in microbial community structure (Figure 5B). Bar plots present the relative abundance of phylum-level taxa (Figure 5C), whereas heatmaps depict differentially abundant genera (Figure 5D). At both the phylum and genus levels, cluster analysis indicated that HNV treatment alleviated intestinal dysbiosis by re-establishing gut microbiota balance. In addition, LEfSe analysis identified differentially abundant taxa between the HNV and DSS groups (Figure 5E-F). Notably, oral HNV treatment increased the relative abundance of *Ligilactobacillus* and *Akkermansia* while decreasing *Clostridium*, a genus associated with depressive behaviors (Figure 5G). It is noteworthy that DSS-challenged animals harbored substantially fewer *Akkermansia* and *Ligilactobacillus* organisms than their HNV-treated counterparts. This pattern points toward a role for HNVs in sustaining gut microbiota homeostasis, which could contribute to the alleviation of chronic colonic inflammation triggered by DSS. More importantly, HNVs modulated the intestinal microbiota by promoting the expansion of beneficial bacterial populations while suppressing pathogenic taxa, which may contribute to the alleviation of IBD-related neuropsychological disorders via regulation of the GBA.

### The effect of gut microbiota changes on immune regulation

**Figure 6:**
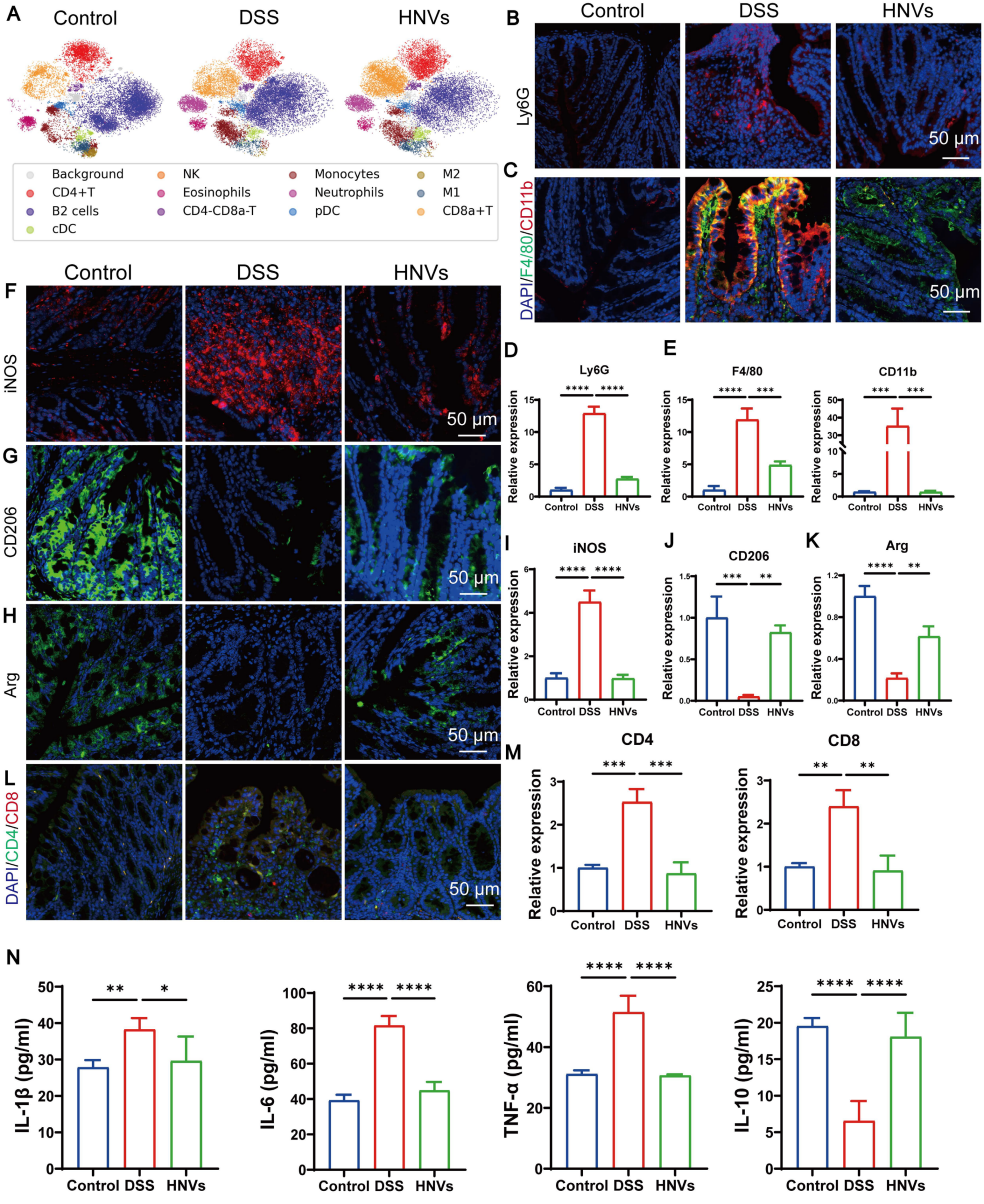
**HNVs regulate intestinal immunity.** (A) Identification and isolation of intestinal immune cells from mice using mass spectrometry flow cytometry. (B) Immunofluorescence staining to detect neutrophil-specific marker Ly6G, Scale bars: 50 μm. (C) Immunofluorescence staining of CD11b/F4-80 in mouse colon tissues, Scale bars: 50 μm. (D) Statistical analysis of the Ly6G immunofluorescence positive area (n = 3). (E) Statistical analysis of F4/80 and CD11b positive cells (n = 3). (F-K) M1 macrophages were labeled with iNOS and M2 macrophages with CD206 and Arg, followed by quantitative analysis (n = 3). Scale bars, 50 μm. (L-M) Immunofluorescence staining of CD4 and CD8 with quantification (n = 3); scale bars, 50 μm. (N) Serum cytokine levels (IL-1β, IL-6, TNF-α, and IL-10) were determined by ELISA (n = 5). Data are presented as mean ± SD. Statistical significance: *p < 0.05, **p < 0.01, ***p < 0.001, **p < 0.0001.

Analysis of the gut microbial community demonstrated that HNVs fostered the expansion of beneficial bacterial populations while concurrently suppressing the propagation of pathogenic species. To investigate the effects of metabolic alterations on intestinal immune responses, colonic immune cells from different groups of mice were profiled using mass cytometry. Classification was based on the positive markers corresponding to each immune cell subset. The DSS group exhibited increased immune cell counts and enhanced neutrophil infiltration compared with the HNV and control groups (Figure 6A), reflecting persistent and significant inflammation. Immunofluorescence staining and quantitative analysis of colon tissues revealed that HNVs significantly reduced the elevation of Ly6G (Figures 6B, D). These findings are consistent with the immune analysis, suggesting that HNV treatment can effectively alleviate inflammation. In the DSS group, there was an increased number of CD11b and F4/80 positive cells (Figure 6C, E), indicating widespread macrophage infiltration in the intestinal tissue. Macrophage subsets were distinguished using specific markers: iNOS for pro-inflammatory M1 macrophages, and CD206 and Arg for anti-inflammatory M2 macrophages. The DSS group exhibited increased M1 macrophages and decreased M2 macrophages, whereas HNV treatment increased M2 (anti-inflammatory) macrophages and reduced M1 (pro-inflammatory) macrophages. These findings suggest that HNVs can reverse the M1 phenotype in intestinal inflammation and promote macrophage polarization toward the M2 subtype (Figure 6F-K). The gut microbiome strongly shapes T cell biology through the production of diverse bioactive small molecules 36. Research indicates that the increased presence of CD4 and CD8 cells can trigger intestinal damage and produce TNF-α, which in turn exacerbates colitis development 37, 38. To further investigate this, immunofluorescence staining was performed on colonic tissues. The average fluorescence intensity of CD8⁺ and CD4⁺ T cells was significantly lower in the DSS group than in the control and HNV groups (Figure 6L-M). HNVs decreased serum pro-inflammatory cytokines and increased the anti-inflammatory cytokine IL-10 (Figure 6N). These results show that HNVs reduce inflammation reactions associated with colitis by regulating intestinal microbiota. HNVs can also regulate the secretion of metabolites, reduce inflammatory reactions, help regulate immune response, and promote the development and maturity of intestinal epithelial cells.

### HNVs regulate 5-HT metabolism via modulation of the gut microbiota

**Figure 7:**
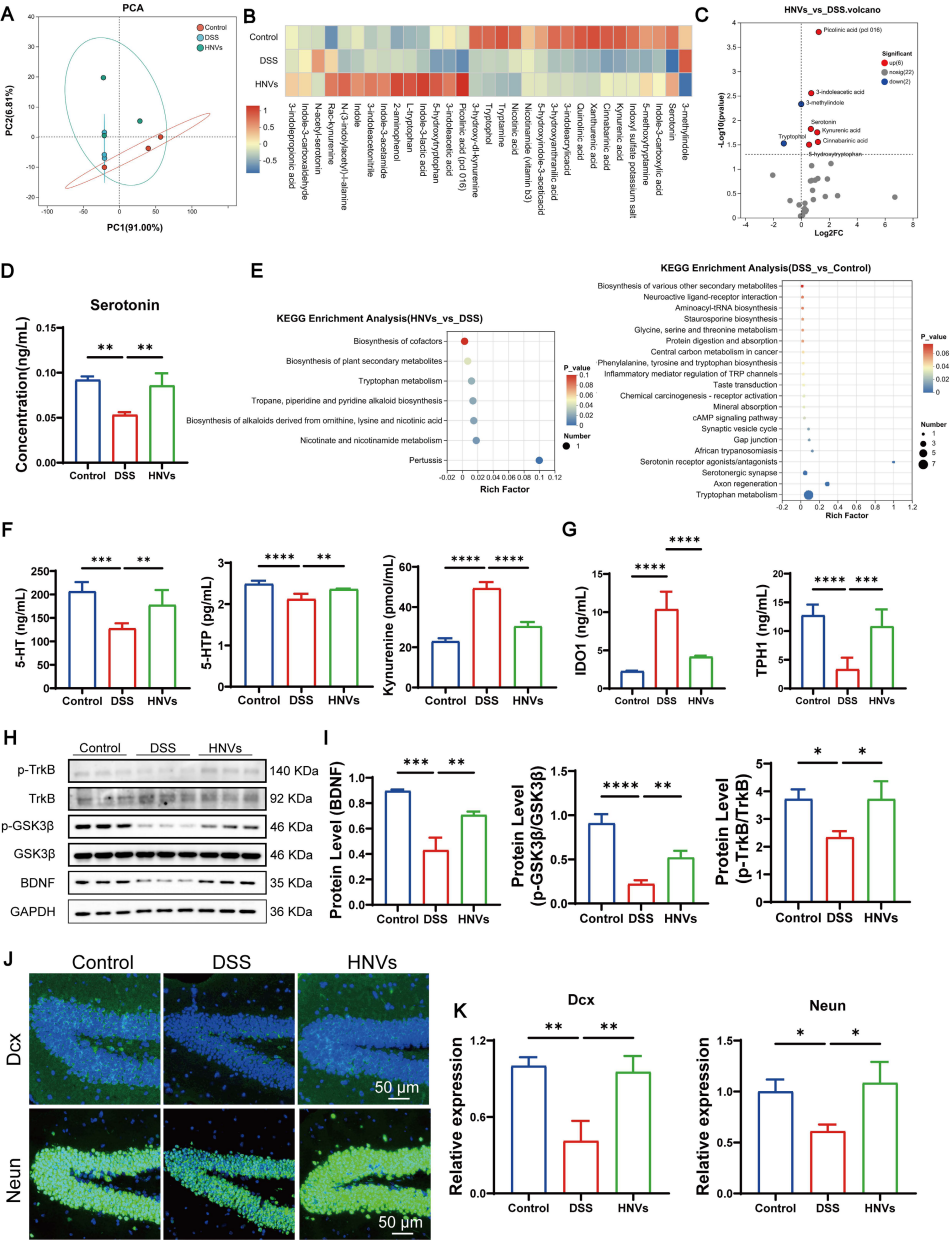
**HNVs alleviate colitis-associated mental disorders by regulating the BDNF/TrkB/GSK3β pathway through Trp metabolism.** (A) PCA of Trp metabolism (n = 3). (B) Cluster analysis of metabolite changes among samples (n = 3). (C) Differential metabolite analysis between the HNVs and DSS groups (n = 3). (D) 5-HT (serotonin) content (n = 3). (E) KEGG pathway enrichment analysis. (F) ELISA detection of serum 5-HT, 5-HTP and Kyn levels (n = 5). (G) ELISA detection of intestinal IDO1 and TPH1 levels (n = 5). (H) WB analysis of p-TrkB, TrkB, p-GSK3β, GSK3β, BDNF, and GAPDH protein expression. (I) Quantification of WB grayscale intensity (n = 3). (J-K) Dcx and NeuN expression in mouse brain tissues was assessed by immunofluorescence staining and quantified (n = 3; scale bars, 50 μm). Results are presented as mean ± SD, with statistical significance defined as ****p < 0.0001, ***p < 0.001, **p < 0.01, *p < 0.05.

To see how HNVs improve colitis-induced mental symptoms through the GBA, we found by 16S rRNA analysis that HNV-treated mice had a marked increase in gut *Akkermansia* and* Ligilactobacillus*. Beneficial intestinal bacteria (such as *Akkermansia* and *Lactobacillus*), can regulate the Trp metabolism pathway, and promote the conversion of Trp to 5-HT. Therefore, we performed targeted metabolomic sequencing of gut Trp. The principal component analysis (PCA) method is used to evaluate the overall metabolic differences between groups and the variability within each group. High reproducibility was observed within each group, whereas significant differences were detected among the normal, DSS, and HNV-treated groups (Figure 7A). Metabolite clustering analysis provided a visual overview of metabolic changes among the groups, revealing notable alterations in metabolites within the HNV-treated group (Figure 7B). Further examination of differential metabolites and their correlations offered insights into how metabolites interact and regulate each other during shifts in biological states. HNV treatment notably increased gut 5-HT levels (Figure 7C-D). KEGG pathway enrichment analysis revealed that DSS primarily affects pathways related to Trp metabolism, axonal regeneration, and serotonergic synaptic processes (Figure 7E).

The Kyn and 5-HT pathways are two main routes of Trp metabolism 39. Under chronic colitis conditions, the Kyn pathway is upregulated, while the 5-HT pathway is suppressed, leading to reduced 5-HT production. In the DSS group, the levels of 5-HT precursor 5-HTP and 5-HT were decreased, while Kyn levels were elevated, indicating the suppression of the 5-HT pathway (Figure 7F). IDO1, a key enzyme, is abundantly expressed in intestinal tissue and controls 95% of free Trp metabolism via the Kyn pathway 8, 40. Peripheral 5-HT synthesis is primarily regulated by TPH1, a rate-limiting enzyme predominantly expressed in intestinal enterochromaffin cells 41. Approximately 90% of peripheral 5-HT is produced by TPH1 in the gut, playing critical roles in regulating intestinal motility, gastric emptying, nutrient absorption, and secretion of gut hormones 42. Certain gut microbiota can promote TPH1 expression in intestinal epithelial cells, thereby enhancing 5-HT synthesis 43. HNVs modulate beneficial gut bacteria to enhance the activity of the key enzyme TPH1 and suppress the expression of IDO1, thereby influencing tryptophan metabolism and promoting the conversion of Kyn to 5-HT (Figure 7G). These results indicate that oral administration of HNVs alleviates IBD-associated depression and anxiety in mice, primarily through GBA mechanisms involving gut microbiota-mediated regulation of 5-HT metabolism.

### Effects of HNVs on brain Trp metabolism in mice with colitis

Intestinal Trp analysis demonstrated that HNV treatment elevated 5-HTP and 5-HT levels in the gut. However, 5-HT cannot cross the BBB. Circulating Trp can be transported across the BBB into the CNS via the large neutral amino acid transporter 1 (LAT1), and then be converted into central 5-HT under the action of enzymes such as tryptophan hydroxylase 2 (TPH2) 44. 5-HTP, as a precursor of 5-HT, can cross the BBB. This is primarily due to its small molecular size and relatively high lipophilicity, characteristics that permit passage through the barrier 45. ELISA showed that in the group treated with HNVs, the average increase in Trp water in serum and brain tissue was significantly increased, and the level of 5-HTP in brain tissue was also markedly increased (Figure S6A-B). These findings suggest that both Trp and 5-HTP can reach the brain via the circulation and cross the BBB. Consistently, expression of TPH2, a key enzyme involved in central 5-HT biosynthesis, was substantially upregulated in the HNVs group (Figure S6C). Together, these results show that the increase in 5-HT levels in brain tissue may not only be due to the enhancement of Trp-dependent synthesis, but also may be relatedto the conversion of itsdirect precursor 5-HTP.

We therefore analyzed brain Trp metabolism. We focused on brain 5-HT and its downstream metabolites. PCA showed good within-group consistency. It also revealed clear differences among the control, DSS, and HNVs groups (Figure S7A). Metabolite clustering analysis illustrated the metabolic patterns unique to each group, showing significant shifts in metabolism following HNV treatment. The most notable change was observed in 5-HT levels (Figure S7B-C). The DSS group exhibited significantly decreased levels of serotonin, indole-3-lactic acid, indole-3-propionic acid, and indole-3-acetic acid relative to the control group. In contrast, HNV treatment increased serotonin and these indole metabolites in the brain (Figure S7D). KEGG pathway analysis indicated that the main changes were related to Trp metabolism, 5-HT signaling, and interactions between signaling molecules (Figure S7E). Together, these findings show that HNVs reshapes brain Trp metabolism in parallel with the rise of intestinal 5-HT. DSS reduced brain serotonin and multiple indole derivatives, whereas HNVs restored these metabolites. The KEGG results support that the major differences converge on Trp metabolism and 5-HT-related signaling pathways. This pattern is consistent with a protective effect of HNVs on the GBA through modulation of a “brain Trp–5-HT axis.”

### HNVs alleviate psychiatric disorders via the BDNF/TrkB/GSK3β pathway

Both BDNF and the TrkB receptor serve critical functions in modulating neural plasticity, adaptive synaptic responses, and emotion-related conduct, particularly within the context of anxious and depressive states 46. Among various factors, 5-HT can activate BDNF expression. Given that HNVs significantly increase serum 5-HT levels, we investigated whether HNVs could enhance BDNF expression in mice. BDNF expression in the hippocampus was significantly reduced in DSS mice, as shown by immunofluorescence staining (Figure S8).

We further explored whether the upregulation of BDNF expression could regulate neuronal growth in IBD mice. We performed Western blotting (WB) to confirm the expression. In the DSS group, the expression of BDNF, TrkB/p-TrkB, and GSK3β/p-GSK3β was downregulated, but HNVs significantly improved these markers in the treated mice (Figure 7H-I). Immunofluorescence staining further revealed that HNVs improved the reduction of immature and mature neurons in the hippocampus of mice (Figure 7J-K). These results suggest that HNVs alleviate IBD-associated anxiety and depression comorbidities by regulating the BDNF/TrkB/GSK3β pathway, promoting neurogenesis, and supporting neuronal growth.

### Biosafety evaluation of HNVs

Both efficacy and safety are critical for translating drug candidates into clinical practice. We evaluated the oral safety of HNVs by giving male C57BL/6J mice 200 μL of PBS or HNVs (10⁹ particles/kg) by oral gavage each day. Blood is collected after treatment for blood routine and blood biochemical testing. Major organs, including the heart, liver, spleen, lung, kidney, and intestine, were harvested for histopathological evaluation. Blood biochemical parameters did not differ significantly among groups, and values in the HNVs group were comparable to controls and remained within normal ranges, supporting good biocompatibility (Figure S9A). Brain tissue H&E staining shows that all group cortex and hippocampus structure are intact (Figure S9B), and no pathological alterations were detected in the main organs (Figure S9C). *In vitro* biocompatibility of HNVs was detected in RAW264.7, HT22, and BV-2 cells. After 24h, the cell survival rate of all concentration groups remained above 80%, indicating that HNVs has good biocompatibility (Figure S10). The above studies show that HNVs have good long-term oral biosafety, no blood toxicity and no tissue toxicity.

## Discussion

This study presents an oral antidepressant vesicle, HNVs, which has been shown to alleviate the pathological symptoms and comorbid psychiatric disorders in colitis mice. This research results show that in the DSS-induced IBD mouse model, the regulation of GBA significantly improved the symptoms of colitis and reduced anxiety and depression-related behaviors.

Over recent years, in response to the comorbidity of mental disorders, the academic community has paid more and more attention to diseases such as severe depression, autism spectrum disorder, anxiety disorder, IBD and schizophrenia 47. People living with IBD face an elevated burden of mental health issues 48. IBD is frequently accompanied by mental health symptoms. About one-third of the patients have anxiety symptoms, and about a quarter of the patients have depressive symptoms. During the illness the burden of these conditions is especially significant: nearly 50% meet the diagnostic criteria for anxiety, while approximately one-third fulfill the criteria for depression 49. There is a two-way relationship between IBD-related inflammation and the development of mental health disorders 50. Psychological factors not only exacerbate disease progression but also lead to a decline in quality of life, increased surgical risks, and higher medical costs. Therefore, recognizing and intervening early in the mental health abnormalities of IBD patients is beneficial for the overall prognosis of the disease 3.

PELNVs is a kind of nanometer-level vesicles with a double-layer membrane structure 51. PELNVs are mainly derived from edible or medicinal plants and possess advantages such as natural bioactivity, low toxicity, low immunogenicity, and easy accessibility at low cost 52. Various PELNVs have been used as biological therapeutic agents and show great potential in disease treatment 53. PELNVs have been recognized as a revolutionary targeted drug delivery system and are considered one of the most promising approaches for disease therapy worldwide 54. As nanoparticles with intestinal immune regulatory functions, PELNVs protect the intestine from inflammatory injury and have attracted broad attention from both clinicians and researchers 55. They preferentially accumulate in the colon and show specific therapeutic effects in colitis and in preserving intestinal homeostasis 56. For example, TDNPs attenuate pro-inflammatory cytokines (TNF-α, IL-6, and IL-1β), thereby promoting intestinal mucosal healing 16, 57. Broccoli derived vesicles can activate the AMPK signaling pathway to maintain intestinal immune balance 58. In addition, nanovesicles derived from garlic, oats, and maca (PELNVs) can improve brain inflammation related diseases through the gut brain axis mechanism 59, 60. Among them, maca derived PELNVs can effectively relieve depression in mice by increasing serum 5 HT levels 61. Consistent with previous studies, our results demonstrate that HNVs effectively alleviate DSS-induced colitis, identifying HNVs as a novel member of the PELNVs family with potent anti-inflammatory activity. The bioactive cargo of HNVs may underlie their therapeutic effects by regulating intestinal immune responses and limiting excessive inflammatory signaling, thereby reducing inflammation in the gut as well as the brain. These findings highlight the therapeutic potential of medicinal PELNVs for neuropsychiatric disorders associated with IBD.

The miRNA in PELNVs can be colonized by intestinal flora and reshape the intestinal microenvironment by regulating intestinal flora. Studies show that the peuMIR2916 p3 of garlic nanovesicles (GaNVs) can specifically promote the growth of *Bacteroides*, thus restoring the balance of intestinal microorganisms 60, and reduces brain inflammation 62. The miRNAgma miR396e from ginger nanovesicles (GNVs) promotes LGG growth by inhibiting LexA, regulating DNA repair, and cell cycle progression 63. GELNs are preferentially internalized by Lactobacillaceae in a lipid-dependent manner and deliver small RNAs that target genes in LGG. Targeting of monooxygenase ycnE by mdo-miR7267-3p enhances indole-3-carboxaldehyde production, which promotes antimicrobial immunity and modulates gut microbial colonization and function 12. HNVs can effectively alleviate DSS-induced chronic colitis. Its mechanism of action may be mediated by the miRNA contained in HNVs, which can regulate the key immune signaling pathway and restore intestinal homeosis. Chronic enteritis is closely related to neuroinflammation and behavioral defects. The miRNA of HNVs may improve related mental disorders by inhibiting systemic inflammation and central inflammatory response, which highlights the functional connection between the intestine and the brain. These findings indicate that HNV-derived miRNAs may represent a promising therapeutic approach for IBD and related neuropsychiatric disorders, although further studies are required to clarify the underlying mechanisms.

To ensure quality consistency of HNVs during the long-term animal study, all batches were produced following a standardized preparation protocol using the same raw materials and processing conditions. Before administration, each batch was subjected to quality assessment, including measurements of particle size, the vesicle count, zeta potential, morphology, and representative compositional markers. Only batches that met the predefined quality-control criteria were used in subsequent experiments. HNV samples were stored as single-use aliquots under standardized conditions to minimize freeze-thaw cycles and ensure batch stability and consistency throughout the study.

HNV dosing was based on particle concentration rather than total protein content. This choice was made to improve the accuracy and reproducibility of vesicle administration. Compared with protein-based normalization, particle number more directly reflects the actual quantity of nanovesicles delivered, whereas protein measurements may be confounded by co-isolated soluble proteins or non-vesicular components introduced during the extraction process. Such variability can compromise the consistency of dosing across samples and experiments. Particle-based quantification, commonly achieved using nanoparticle tracking analysis, enables more precise standardization by ensuring that comparable numbers of vesicles are administered across experimental groups. This strategy has been increasingly recommended in the extracellular vesicle field as a means to enhance experimental reproducibility and facilitate cross-study comparisons. Moreover, given the intrinsic heterogeneity of HNVs and the potential variability in protein cargo depending on source material and isolation procedures, normalization based on protein concentration may not accurately represent vesicle abundance or biological activity. In contrast, particle-based dosing provides a more consistent and biologically relevant metric for evaluating therapeutic effects. Nevertheless, we acknowledge that each quantification method has its limitations, and future studies integrating multiple normalization strategies (e.g., particle number, protein content, and functional assays) may further improve the standardization of nanovesicle-based therapies.

GBA elaborates on the two-way communication between CNS and the digestive tract, involving neurotransmitters, neuroimmune signals, neuroendocrine pathways and sensory nerves. More and more evidence emphasizes the key role of GBA in depression and other related diseases 64-67. IBD and its mental comorbidity are often regarded as GBA diseases. Accordingly, the bowel can shape neurological function utilizing the intrinsic enteric neural network, neuroendocrine signaling via the hypothalamic-pituitary-adrenal axis, and various immune-associated conduits 68. Intestinal flora disorders and their metabolites can cause chronic intestinal inflammation and aggravate neuroinflammation through GBA 69. When the intestinal barrier is damaged, microbial products like LPS and cytokines can enter the blood and cause entensive inflammatory reactions 70, 71. Inflammatory factors in the blood can pass through the BBB into the hippocampus and other areas. This may lead to neuroinflammation and neuron damage, and increase the risk of mental disorders 4. Under inflammatory conditions associated with IBD, levels of factors such as LPS and IL-1β are elevated, which may disrupt the BBB, allowing inflammatory signals to enter the brain and potentially induce neuroinflammation, thereby contributing to IBD-associated neuropsychiatric disorders. In this study, HNVs significantly reduced inflammatory factors in both serum and brain tissue. Gut microbiota dysbiosis may be one of the key triggers of chronic inflammation and is closely related to a variety of neuropsychiatric diseases 72. *Lactobacillus* and *Akkermansia* were enriched in treated mice relative to the DSS group, suggesting a potential role for these microbes in the antidepressant- and anxiolytic-like effects of HNVs.

IBD and intestinal inflammatory disorders, such as UC, are closely related to intestinal microbiome disorders and immune dysfunction. Trp is an important metabolite in regulating gut immune function and has a significant regulatory effect on intestinal inflammation by influencing the metabolism and signaling of 5-HT in the gut 73. The primary site of Trp conversion to 5-HT is the intestine, and it is also the main source of peripheral 5-HT. Gut microbiota play a key role in regulating Trp metabolism 74. As an essential dietary amino acid, Trp generates metabolites that act as neurotransmitters and signaling molecules in various physiological processes. Among these, 5-HT regulates sleep, mood, appetite, and cognitive function in the CNS. Abnormal levels of 5-HT are associated with depression, anxiety and other mental disorders, and 5-HT is crucial to gastrointestinal function. In the gastrointestinal tract, 5-HT regulates motility, secretion, and immune function. Its level imbalance is related to intestinal stress syndrome, diarrhea, constipation and other diseases, and interacts with intestinal flora 75. At the same time, we acknowledge that microglia-mediated neuroinflammation also plays a critical role in IBD-related neuropsychiatric symptoms. In this study, we observed that HNV treatment can reduced the activation of microglia, indicating that its anti-inflammatory effects on the brain may also help improve behavior. These findings indicate that both serotonergic regulation and neuroinflammation may be involved in the therapeutic effects of HNVs. Although this study emphasizes the role of 5-HT, future research still needs to further explore the interaction between the neuronal mechanism and the immune mechanisms in GBA.

Synaptic plasticity is synergistically regulated by 5-HT and BDNF, with 5-HT enhancing BDNF expression 76. The development of various neuropsychiatric disorders is modulated by key mediators, including 5-HT and BDNF, which play critical roles in conditions such as hepatic encephalopathy associated with cirrhosis. BDNF, a key neurotrophic factor, promotes neuronal survival and growth and is critical for neuroplasticity as well as learning and memory 77. Upregulation of BDNF expression in the hippocal region of the brain tissue of chronic colitis mice treated with HNVs. Subsequently, BDNF combines with its specific receptor TrkB with high affinity. This combination triggers the signaling pathway of regulating synaptic plasticity, improving learning memory and helping to reduce depression. Zhizi-Houpo Decoction alleviates depression-like behavior in CUMS mice by activating the BDNF/TrkB/CREB pathway, thereby promoting neurogenesis in the hippocampus 78. Maca, by boosting 5-HT levels, regulates the GTP-CDC42/ERK pathway, enhances the expression of BDNF, a key neuroplasticity regulator, and subsequently activates the TrkB/p-AKT signaling pathway 61. BDNF is highly expressed in the brain areas related to emotional regulation, such as the prefrontal lobe and the hippocampus region. It activates TrkB receptors, thereby triggering the PI3K/Akt signaling pathway and inhibiting the excessive activation of GSK3β. This process can inhibit neuroinflammation, maintain synaptic stability and improve mood 79, 80. The overactivation of GSK3β is the pathological basis of various emotional disorders. Studies show that chronic inflammation can lead to a significant decrease in peripheral and central BDNF levels, while increased GSK3β activity is closely related to neuronal apoptosis, synaptic dysfunction and depressive behavior 79. In the brain tissue of mice with chronic colitis, HNVs increased the expression levels of BDNF as well as phosphorylated TrkB and GSK3β. HNVs regulate Trp levels and increase 5-HT levels by modulating the gut microbiota, thereby alleviating inflammation-associated neuropsychiatric disorders. This effect is mediated through the BDNF/TrkB/GSK3β signaling pathway.

There are still many limitations in this study. The antidepressant and anxiety effects produced by HNVs may be attributed to the presence of some antidepressant or anxiety compounds in the vesicle. Prior multi-omics investigations by our team into HNVs-mediated amelioration of acute colitis have demonstrated that these vesicles encapsulate diverse bioactive constituents, encompassing proteins, lipids, nucleic acids, and secondary metabolites. HNVs contain various lipids and 2,932 miRNAs. They are mainly composed of organic oxides, carboxylic acids and their derivatives, fatty acyls, propanol lipids, and flavonoids 15. However, the key factors of HNVs anti-depression and anxiety have not been fully studied. HNVs contains a variety of lipid molecules, which play a key role in vesicle formation, stability and interaction with cells. Exosome-like nanovesicles have a very diverse lipid composition. Lipid composition and abundance may vary with extraction methods and growth conditions of honeysuckle. This variability presents challenges for achieving standardization and large-scale production. miRNAs, as crucial bioactive molecules within HNVs, regulate the expression of genes related to depression by binding to target mRNAs. Although certain miRNAs show promising therapeutic potential, their vulnerability to degradation, interactions with multiple targets, and potential biosafety concerns pose significant barriers to their direct pharmaceutical development. While the therapeutic effects of HNVs have been demonstrated, important mechanistic questions still remain unanswered. The specific components of HNVs responsible for regulating the gut microbiota and alleviating depression need further exploration. Future research will focus on identifying the bioactive components of HNVs with therapeutic potential, as well as clarifying their target cell specificity and mechanisms of action to address these unresolved aspects.

## Conclusion

In summary, our findings suggest that oral HNVs help maintain colonic epithelial barrier integrity, support BBB function, regulate systemic immune responses, and modulate gut microbiota composition. HNVs regulate the intestinal microbiota by promoting the expansion of beneficial bacterial populations while suppressing pathogenic taxa, thereby enhancing TPH1 expression and reducing IDO1 expression. This modulation of Trp metabolism promotes the conversion of Kyn into 5-HT. Trp and 5-HTP in the gut cross the BBB via the bloodstream, thereby enhancing brain 5-HT levels. This modulation influences the BDNF/TrkB/GSK3β pathway, leading to improvements in anxiety and depression comorbidities associated with IBD. The altered MGBA plays a key role in alleviating psychiatric disorders associated with colitis. This study combines pharmacology, microbiology, nanobiology, and other interdisciplinary approaches, and, grounded in the GBA theory, uncovers the mechanism through which HNVs alleviate psychiatric disorders related to IBD. This offers a scientific foundation for developing safe, natural, and orally deliverable intervention strategies with TCM nanovesicles. By promoting the clinical translation of HNVs, this study also offers new insights into the theoretical framework and application potential of medicinal PELNVs.

## Experimental Methods

*Generation and physicochemical characterization of HNVs:* Honeysuckle (100 g; purchased from Tongjuntang) was mixed with 1 L of PBS and homogenized using a preset fruit-processing program for 3 min. The resulting mixture was filtered to remove insoluble residues, yielding clarified honeysuckle juice for further processing and characterization. The honeysuckle juice was centrifuged at 4 °C, 10,000 rpm for 60 min, repeated three times, and the supernatant was collected, and the filter membrane is 0.45 (Millipore MultiScreen®). Pour the filtered liquid into the crossflow filtration system purification instrument and concentrate it to 50mL through a hollow fiber column of 750 KD (TMP<4 Mpa, AutoTFF075-M; Inscinstech, Suzhou, China). Size and zeta potential characterization relied on the Zetasizer Nano ZSE (Malvern Instruments, United Kingdom). For determining particle concentration, a NanoCoulter counter provided by Resun Technology Co., Ltd. (Shenzhen, China) was utilized.

*Preparation of herbal decoction***:** 100 g honeysuckle is fried in 1000mL of water according to the TCM frying method, and prepare the broth HD.

***Stability in simulated gastrointestinal fluids:*** The stability of HNVs in simulated gastrointestinal fluids was assessed by incubating the particles with simulated gastric and intestinal fluids at 37°C for 0, 6, 12, and 24 hours. Particle size distribution was evaluated at each time point using DLS (Zetasizer Nano ZSE). In order to further verify the existence of exocrine-like vesicles, the 24-hour sample was treated with lysis reagents (hydrogen peroxide) 81, so as to obtain HNVs (HNVs- H_2_O_2_) with damaged membrane structure. Thereafter, TEM coupled with DLS was employed to re-examine particle counts along with the distribution of particle dimensions.

*Hemolysis experiment:* Following isoflurane anesthesia (RWD Life Science Co., Ltd, Shenzhen, China), whole blood was harvested via the orbital venous sinus and immediately placed in anticoagulant-coated collection vessels. After centrifuing for 15 min under the condition of 1000g 4 degrees, take the supernatant and isolate the red blood cells (repeat 2-3 times until the supernatant is transparent), and the last supernatant is the red blood cell suspension. Prepare 2% blood cell suspension. Different concentrations of HNVs were mixed with 500 microliters of a two percent red blood cell suspension and incubated at 37 °C for three hours. Centrifuge the mixture, take the supernatrum and transfer it to the 96-hole plate to record the absorbance at 540nm to calculate the hemolysis rate.

*Experimental animals:* Male C57BL/6 mice, aged 6 to 8 weeks, were procured from Hangzhou Medical College (accredited under SYXK [Zhe] 2024-0008). All animals were maintained under SPF-grade husbandry conditions, and investigations were executed in alignment with ethical guidelines authorized by the Zhejiang Provincial Laboratory Animal Center's Animal Welfare and Ethics Committee (ZJCLA-IACUC-20011248). Randomly divided the mice into 9 groups (n = 10): Control, DSS, DSS+HNVs-L, DSS+HNVs-M, DSS+HNVs-H, DSS+Flu, DSS+5-ASA, DSS+HNVs-H_2_O_2_ and DSS+HD. Colitis was induced by administering 2% (w/v) DSS (molecular weight 36,000 to 50,000; MP Biomedicals, USA) in drinking water for 7 days, followed by a 7-day recovery period. After the first cycle, Control and DSS group were gavaged daily with 200 μL of PBS, DSS+HNVs-L (HNVs-L, 1 × 10^9^ particles/kg), DSS+HNVs-M (HNVs-M, 1 × 10^10^ particles/kg), DSS+HNVs-H (HNVs-H, 1 × 10^11^ particles/kg), DSS+Flu (Flu, 10 mg/kg), DSS+5-ASA (5-ASA, 100 mg/kg), DSS+HNVs- H_2_O_2_ (HNVs- H_2_O_2,_ 100 mg/kg), and DSS+HD (HD, 2 g/kg). Body weight were monitored daily. On day 57, behavioral tests were performed.

*Histopathological analysis:* After euthanasia, brain and colon tissues were harvested and fixed in 4% paraformaldehyde for H&E staining. Tissue sections were then imaged using a Leica microscope. The scoring criteria were based on each field of view, including the degree of inflammatory cell infiltration (0-3 points), the depth of tissue damage (0-3 points), the damage to crypt structure (0-4 points), and the ratio of damaged area (0-4 points).

*Small animal imaging:* C57BL/6 male mice, aged 6-8 weeks, randomly divided into three groups (Control, DSS, DSS+HNVs), 7 in each group. A chronic colitis model was established. At the end of the fourth treatment cycle, DiR- and PKH26-labeled PBS was administered to control and DSS mice, while HNV-treated mice received DiR- and PKH26-labeled HNVs orally. Fluorescence imaging *in vivo* was performed at 0, 2, 4, 6, 8, 12, and 24 hours using an IVIS Spectrum system (PerkinElmer, Waltham, MA, USA). After imaging at each time point, mice were euthanized and major organs were collected for *ex vivo* imaging.

*Behavioral experiments:* All behavioral tests are carried out in the light phase. Behavior analysis is carried out by researchers who are not aware of the experimental conditions.

*Open field test:* OFT is used to analyze spontaneous exploration activities and assess the degree of anxiety. The mouse is placed in the OFT center, illuminated under dim light, and the tracking software (SMART 3.0, Panlab) is used to automatically record the movement trajectory of the mouse within 5 min.

*Elevated plus maze:* The maze consists of a central platform, two open arms and two closed arms, and the installation is 50 cm above the ground. Place the mouse on the apparatus and allow it to move freely for 5 min, while recording its movement trajectory and the time spent in the open and closed arms.

*Tail suspension test:* The animals underwent tail suspension by means of adhesive tape, and the nose-to-floor vertical distance was fixed at 25 cm. The test lasted 6 min, and immobility time during the last 4 min was recorded for analysis.

*Forced swim test:* Each mouse is placed individually into a cylindrical container filled with water (diameter: 10 cm; depth: 30 cm) maintained at 23-25°C. To ensure the mice were unable to use either their tails or back legs as props, the water depth was appropriately regulated. Use tracking software to automatically record 6-min tests. Clean and deodorize the cylinder before each test. During the test, the environment should be quiet and the lighting should be consistent to eliminate any potential interference from the environment or human factors.

*Cellular Uptake:* Remove the frozen storage tube containing RAW264.7 cells from the liquid nitrogen tank and place it in a 37 °C water bath to thaw. After thawing, transfer the cells to the centrifuge tube and centrifuge at 800 rpm for 3 min. Remove the cryoprotectant and add 1 mL of RPMI-1640 (Hangzhou Yangming Biotechnology Co.,Ltd) complete culture medium to the cells. Gently pipette to achieve a uniform cell suspension. Transfer the cells into a culture dish, then incubate at 37 °C in a humidified atmosphere containing 5% CO₂. Inoculate the cells into the confocal dish with a density of 104 cells/mL. When the cells grow to about 60%, add PKH26-labeled HNVs according to the time gradient of 0, 6, 12 and 24h, and apply 37 °C. After the application, wash the cells with PBS 2-3 times, add 1μL Hoechst and apply them at 37 °C for 20-30 min. After cleaning with PBS, use SP8 laser confocal instrument (Leica, Wetzlar, Germany) for testing. Cell counting and sorting were performed using a NovoCyte Advanteon V6BSR3 flow cytometer (Agilent Technologies).

*Cell viability and toxicity analysis:* Raw264.7 cells were seeded into a 96-well plate. Each group contained six wells, and seven groups were set up in total: blank control, and HNVs at concentrations of 10^2^, 10^4^, 10^6^, 10^8^, 10^10^, and 10^12^ particles/mL. Following 24 hours of incubation, 100 µL of culture medium and 10 µL of CCK-8 solution were added to each well. Incubate the culture plate at 37 °C in the dark for 30 min, then measure the absorbance at 450 nm to assess cell viability.

*ELISA:* Serum LPS levels were measured using a mouse LPS ELISA kit from Jianglai Bio. Serum IDO1, TPH1, TPH2, 5-HTP, S100β, Trp and 5-HT levels were measured with the corresponding mouse ELISA kits. The concentrations of IL-1β, IL-10, IL-6, and TNF-α in brain, serum and intestinal tissues were determined using mouse-specific ELISA kits. Absorbance was measured at 450 nm with an Epoch microplate reader (BioTek, USA).

*16S rRNA:* Fecal samples were processed for total microbial DNA extraction using the FastPure Stool DNA Isolation Kit. Purified PCR products were used for library preparation with the NEXTFLEX Rapid DNA-Seq Kit, followed by sequencing on an Illumina NextSeq 2000 system. After sequencing, paired-end reads were filtered and merged. The sequences were then denoised with the DADA2 plugin to generate ASVs. Alpha- and beta-diversity analyses were conducted using ASV representative sequences paired with quantitative abundance profiles. Taxonomic profiles were summarized by relative abundance to identify different phyla and genera.

*Tryptophan-targeted metabolism:* Fecal and brain tissue samples (25 mg each) were accurately weighed. The residue was reconstituted in 100 μL of acetonitrile-water, sonicated at 5 °C for 15 min (40 kHz), and then centrifuged at 13,000 rcf for 15 min at 4 °C. The final supernatant was collected for LC-MS/MS analysis. Metabolomic profiling was performed on a Nexera LC-40 system coupled with a QTRAP 6500+ mass spectrometer. Metabolite concentration data were analyzed via the Majorbio Cloud Platform.

*Immunofluorescence:* After the behavioral experiment, the mice were anesthetized with isoflurane and the heart was perfused with 4% paraformaldehyde (PFA). Remove the brain and intestinal samples and let it stay overnight in 4% PFA at 4 °C and dehydrated stepwise. Cut the brain and intestinal samples into continuous coronal frozen slices, and then close them after dewaxing, antigen repair and 10% goat serum. Tissue sections were exposed to primary antibodies at 4 °C overnight, including claudin-1 (PTG, 1:200), ZO-1 (PTG, 1:200), CD206 (PTG, 1:300), iNOS (PTG, 1:200), F4/80 (Servicebio, 1:300), CD11b (Abways, 1:200), CD4 (Abcam, 1:200), CD8 (Abcam, 1:200), DEX(PTG, 1:200), Neun (Abways, 1:200) and BDNF (1:200, Abcam). Apply 4 °C overnight, use PBST to configure the second antibody according to the dilution ratio of 1:200, DAPI dye the nucleus, and store the finished fluorescent film in 4 degrees away from light.

*Western blot:* After cardiac perfusion, the mouse brain tissue was removed and the protein was extracted according to the steps of the whole protein extraction kit (KeyGEN BioTECH). Protein samples were denatured by boiling and subsequently resolved on 10% SDS–PAGE gels. Detection of target proteins was accomplished by applying the following primary antibodies: anti-BDNF from Abcam (1:1000), anti-GSK3β (1:1000) and anti-p-GSK3β (1:1000) from Cell Signaling Technology, anti-TrkB from Proteintech (1:1000), anti-p-TrkB from Cell Signaling Technology (1:1000), and anti-GAPDH from ABclonal (1:10000). The secondary antibody employed was anti-rabbit IgG, obtained from Cell Signaling Technology. We detected the protein bands using an ECL substrate. The images were captured with a qTouch WB Imager from RWD Life Science in Shenzhen, China.

*Serum biochemical analysis:* After treatment, mouse serum and whole blood were collected. Routine blood tests were performed using a TEKON automatic hematology analyzer (TEKON Technology). The automatic biochemical analyzer was used to measure serum biochemical parameters, including AST, ALT, BUN, and CREA.

***Statistical analysis:*** The data were represented as the mean and standard deviation (SD). One-way analysis of variance (ANOVA) and Tukey’s post-hoc test were performed to evaluate statistical significance using GraphPad Prism software (GraphPad v10.4.1, USA). A p-value of < 0.05 was considered significant.

## Supplementary Material

Supplementary figures.

## Figures and Tables

**Figure 8:**
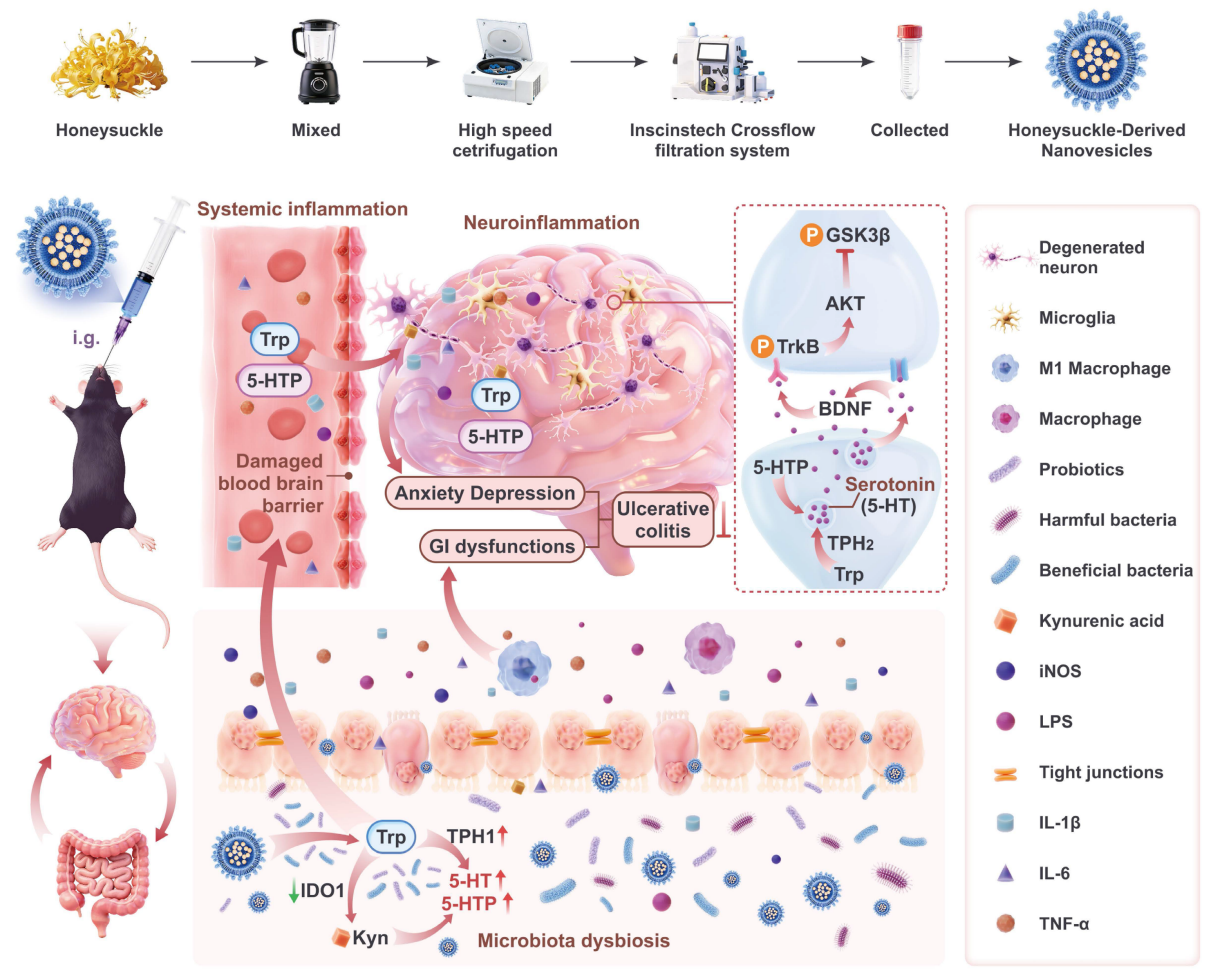
**Schematic illustration of HNVs alleviating IBD-associated neuropsychiatric disorders via the gut–brain axis.** HNVs is separated by ultra-fast centrifugation combined with tangenal flow filtration technology. HNVs interacts with intestinal flora after oral gavaged to alleviate IBD-related mental disorders by regulating GBA. DSS-induced chronic colitis will destroy the balance of intestinal flora and damage the gut barrier, resulting in increased levels of systemic LPS and pro-inflammatory factors. These inflammatory factors will destroy the integrity of BBB and cause neuroinflammation. HNVs can repair intestinal barrier damage, reduce systemic inflammatory response, and repair BBB damage, thus preventing inflammatory factors and LPS from entering the hippocampus and causing neuroinflammation. HNVs also increased the abundance of beneficial intestinal microbiota, improved the intestinal immune microenvironment, and regulated Trp metabolism by upregulating TPH1 and downregulating IDO1, thereby shifting Kyn metabolism toward the 5-HT biosynthetic pathway. HNVs enhance 5-HT levels in brain tissue by regulating the levels of 5-HTP and Trp in both the blood and brain, thereby increasing TPH2 levels in the brain, resulting in increased BDNF expression and TrkB signaling pathway activation. This pathway restrains excessive GSK3β activity, supports neurogenesis and neuronal development, and ultimately alleviates IBD-associated emotional disturbances. This figure was generated using biorender, blender, and figma in combination.

## Data Availability

The datasets used and/or analysed during the current study available from the corresponding author on reasonable request.

## Abbreviations

CD: Crohn's disease
DSS: Dextran sulfate sodium
DLS: Dynamic light scattering
ELISA: Enzyme-linked immunosorbent assay
EPM: Elevated plus maze
FST: Forced swim test
GaNVs: Garlic nanovesicles
GNVs: Ginger nanovesicles
GBA: Gut-brain axis
HNVs: Honeysuckle-derived nanovesicles
IBD: Inflammatory bowel disease
Kyn: Kynurenine
LPS: Lipopolysaccharide
PAGE: Polyacrylamide gel electrophoresis
PCoA: Principal coordinate analysis
5-HT: Serotonin (5-hydroxytryptamine)
S100β: S100 calcium-binding protein β
SPF: Specific pathogen-free
TST: Tail suspension test
TCM: Traditional Chinese medicine
Trp: Tryptophan
TEM: Transmission electron microscopy
UC: Ulcerative colitis
WB: Western blot
