# Supplementary material for: Honeysuckle-derived nanovesicles alleviate inflammatory bowel disease-associated neuropsychiatric disorders via the gut–brain axis
Source: Theranostics. 2026 Jun 10;16(13):7419–41. doi: 10.7150/thno.132241 (PMC13295170; doi:10.7150/thno.132241)
Supplement: Supplementary file 1 — Supplementary figures. [file thnov16p7419s1.pdf]

Supporting Information

Honeysuckle-Derived nanovesicles alleviate inflammatory bowel disease–associated neuropsychiatric disorders via the gut–brain axis

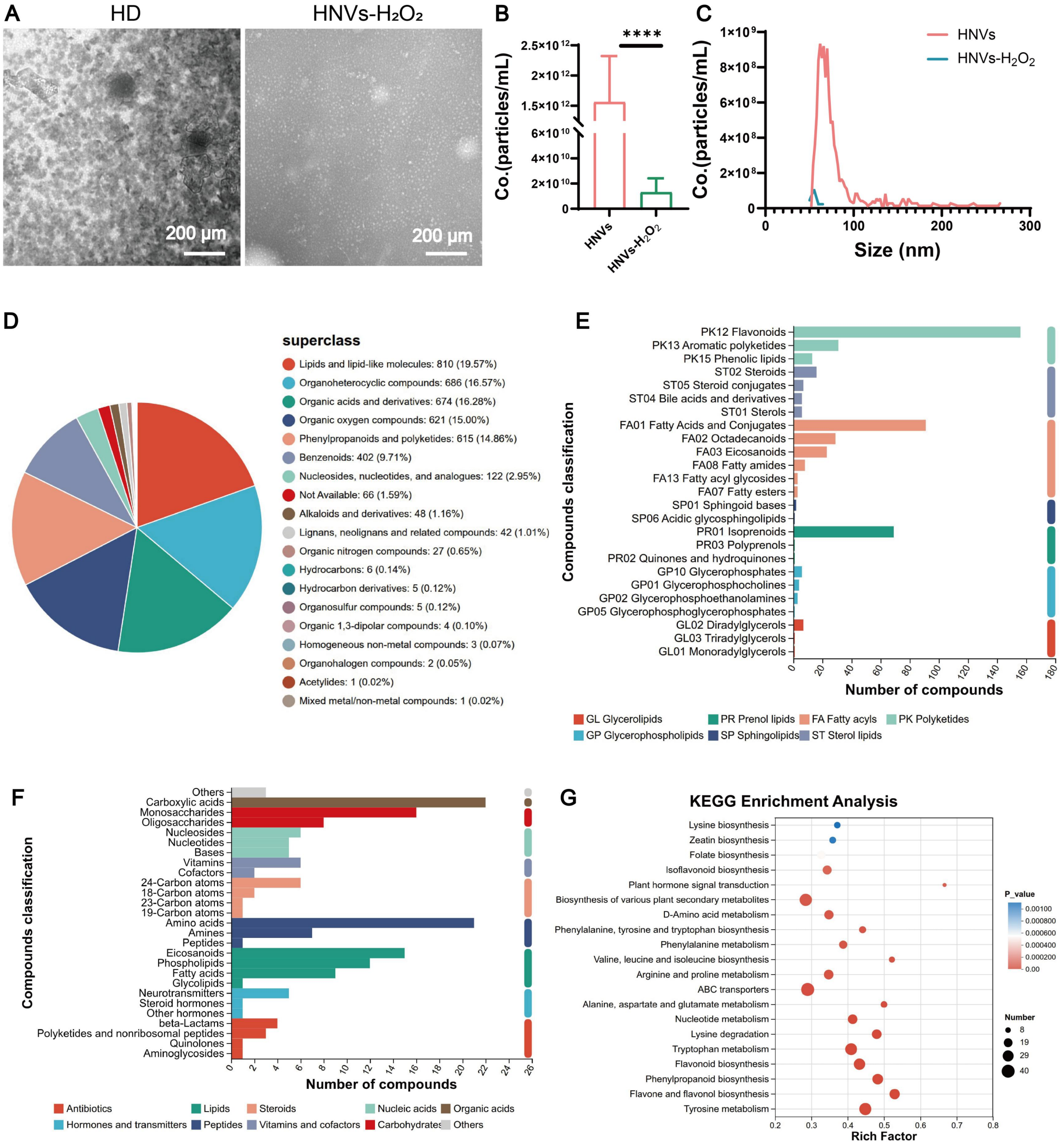

Figure S1. (A) TEM observation of the morphology of HD and HNV-H_2O_2. (B-C) NanoCoulter counter analysis of the particle counts of HNVs and HNVs-H_2O_2 (n = 3). (D) Classification of compounds identified in HNVs. (E) Classification of lipid compounds identified in HNVs. (F) Classification of bioactive compounds identified in HNVs. (G) KEGG pathway enrichment analysis of compounds identified in HNVs. Data are expressed as mean ± SD, No statistical significance (ns) $p \geq 0.05$, *$p < 0.05$, **$p < 0.01$, ***$p < 0.001$, ****$p < 0.0001$

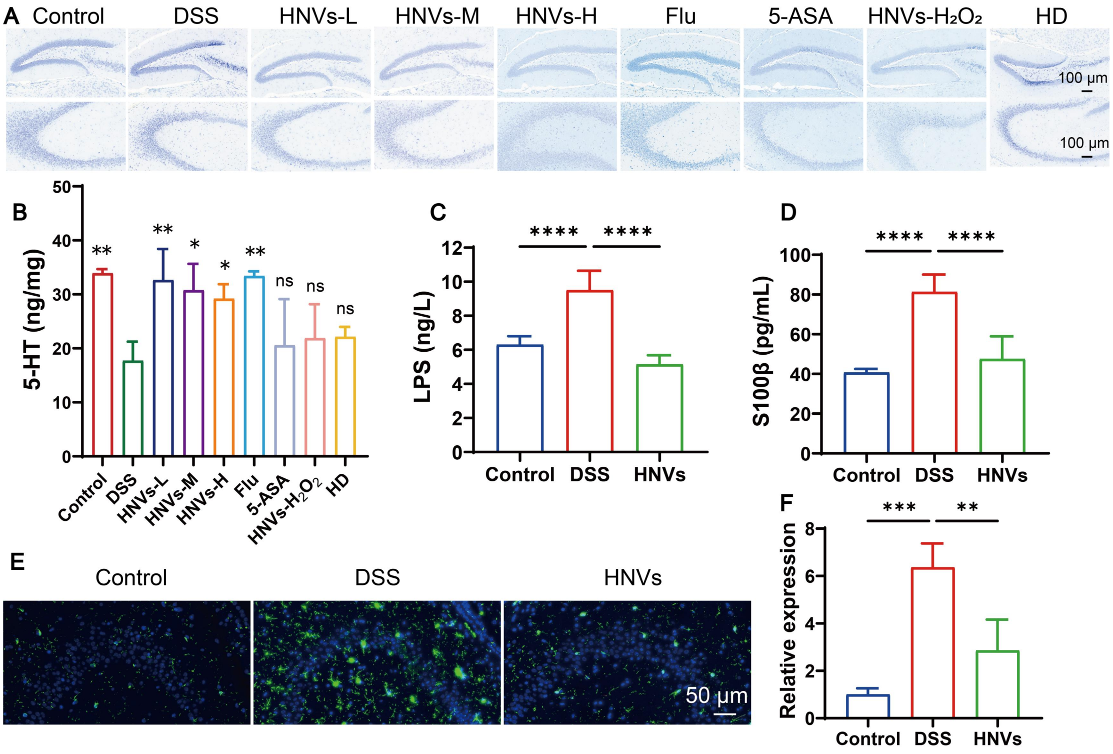

Figure S2. (A) Nissl staining of brain sections from each group of mice. Scale bar is 100 μm (B) 5-HT levels in mouse brain tissue were measured by ELISA (n = 3). (C) Levels of LPS in mouse serum (n = 5). (D) Serum S100β levels in mice (n = 5). (E-F) Immunofluorescence detection of the microglial marker IBA-1 in brain tissue and its quantification (n = 3). Data are expressed as mean ± SD, No statistical significance (ns) $p \geq 0.05$, *$p < 0.05$, **$p < 0.01$, ***$p < 0.001$, ****$p < 0.0001$

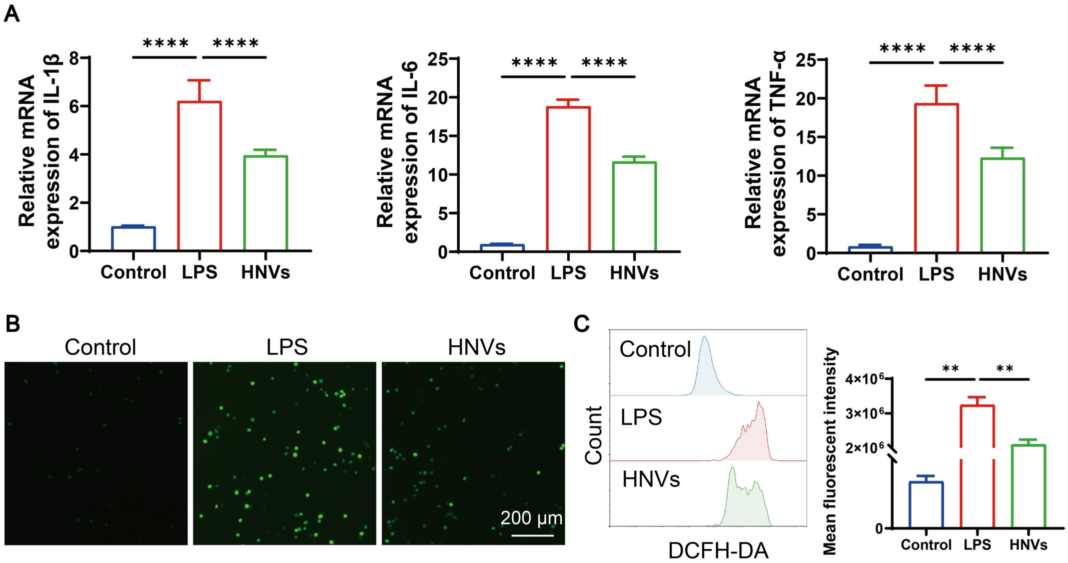

Figure S3. In vitro anti-inflammatory effects of HNVs. (A) IL-1β, IL-6, and TNF-α mRNA expression levels in RAW264.7 cells following different treatments (n = 6); (B) DCFH-DA staining for ROS generation in RAW64.7 cells after different treatments, Scale bar, 200 μm; (C) Flow cytometric quantification of ROS detection and fluorescence intensity in RAW264.7 cells (n = 3). Data are expressed as mean ± SD, No statistical significance (ns): $p \geq 0.05$; *$p < 0.05$; **$p < 0.01$; ***$p < 0.001$; ****$p < 0.0001$.

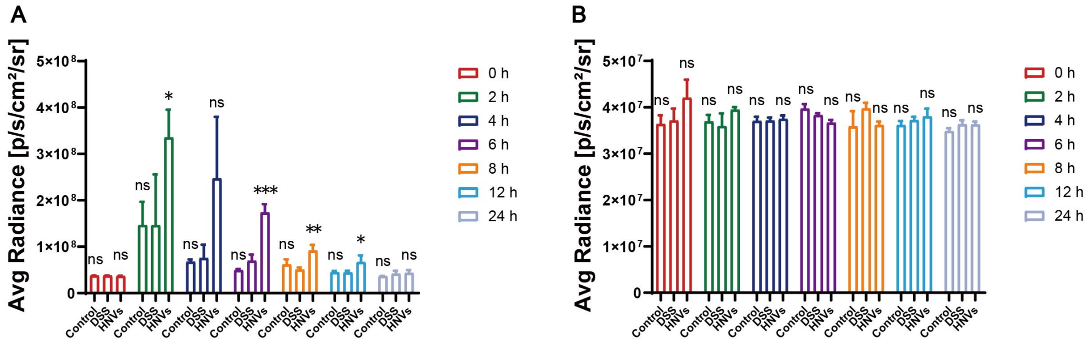

Figure S4. (A) Quantitative analysis of average fluorescence intensity in the colon at different time points (n = 3). (B) Analysis of average fluorescence intensity in liver tissue at different time points (n = 3). Data are expressed as mean ± SD, No statistical significance (ns): $p \geq 0.05$; $^{*}p < 0.05$; $^{**}p < 0.01$; $^{***}p < 0.001$; $^{****}p < 0.0001$.

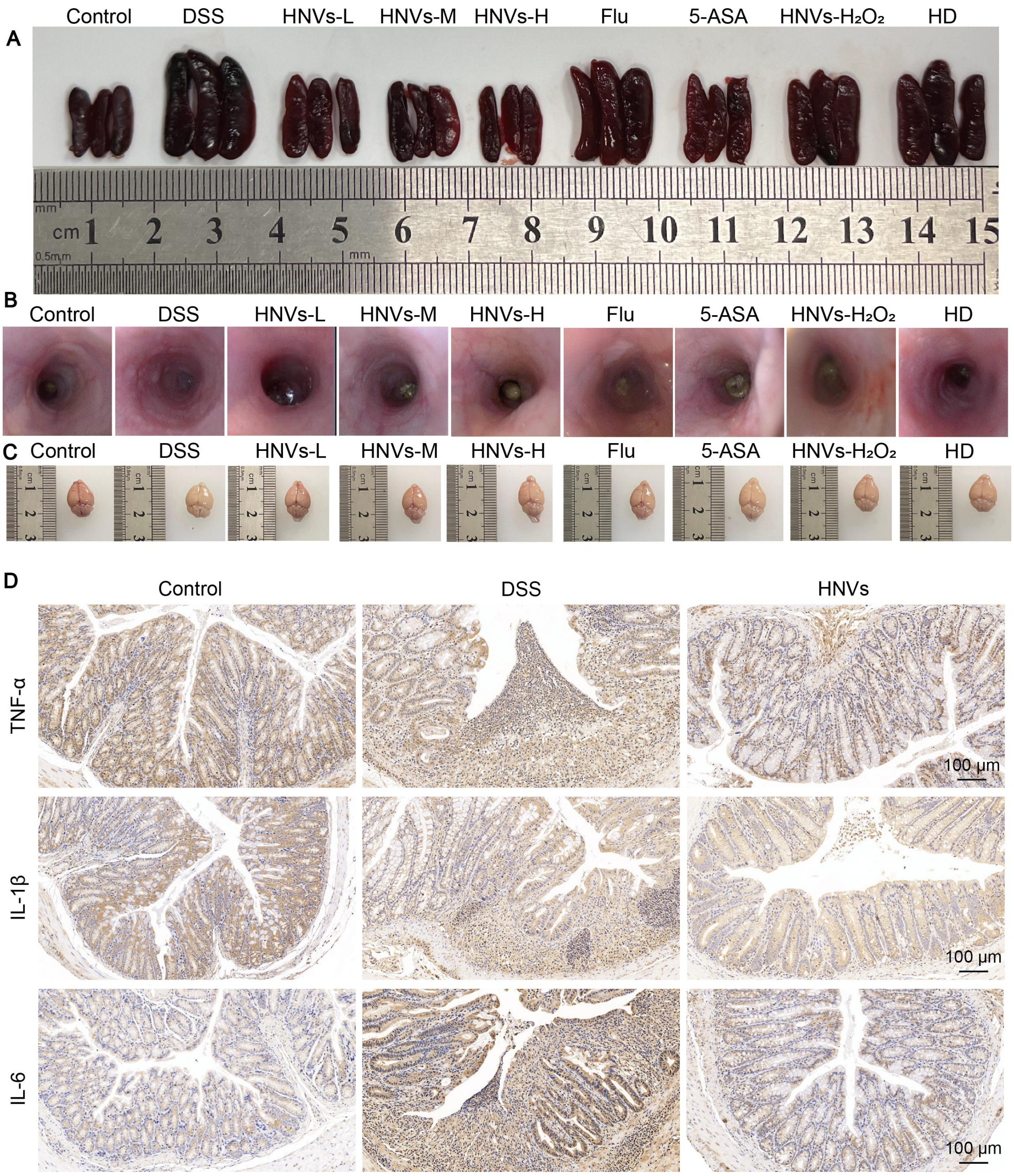

Figure S5. (A) Anatomical images of mouse spleens from each group (n = 3). (B) Colonoscopy images of mice in each group. (C) Gross anatomy of brain tissues from each group. (D) Immunohistochemical staining for IL-1β, IL-6, and TNF-α; Scale bar: 100 μm.

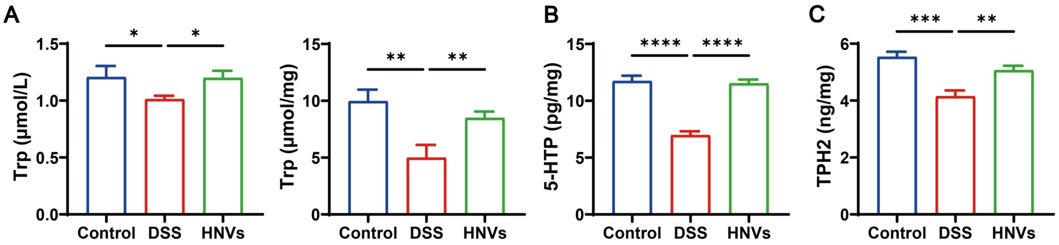

Figure S6. (A) Trp levels in serum and brain tissue (n = 3); (B) 5-HTP levels in brain tissue (n = 3); (C) TPH2 levels in brain tissue (n = 3). Data are shown as mean ± SD, No significance (ns) $p \geq 0.05$, *$p < 0.05$, **$p < 0.01$, ***$p < 0.001$, ****$p < 0.0001$

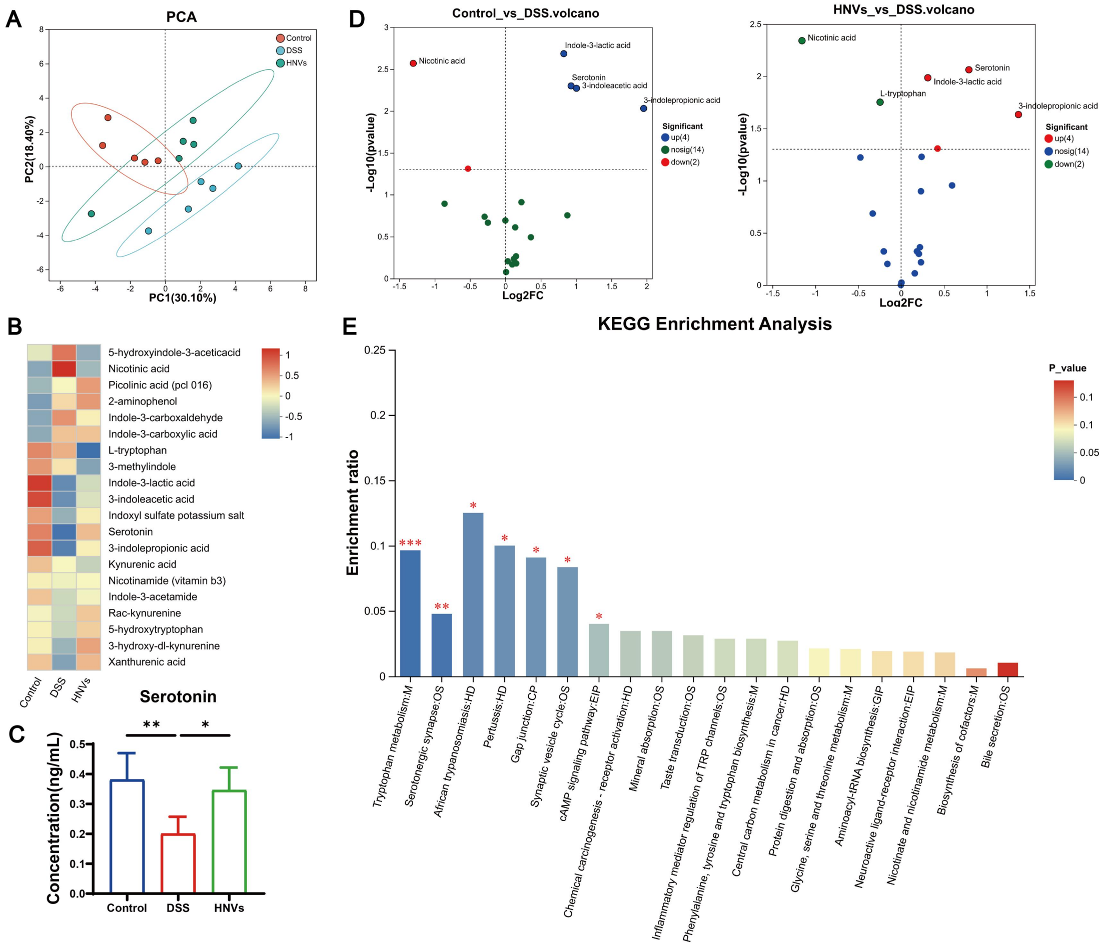

Figure S7. HNVs regulate tryptophan metabolism in the brain. (A) PCA analysis of samples from each group (n = 5). (B) Cluster analysis of metabolite levels in each group; (C) Serotonin levels (n = 5). (D) Comparison of differential metabolites between the DSS and control groups, as well as between the DSS and HNVs-treated groups. (E) KEGG pathway enrichment analysis. Data are shown as mean ± SD, **p* < 0.05, ***p* < 0.01.

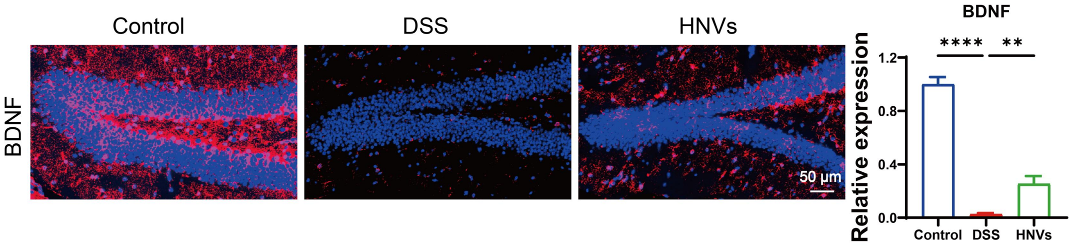

Figure S8. Immunofluorescence detection of BDNF in mouse brain tissue (n = 3), Scale bar: 50 μm. Data are shown as mean ± SD, No significance (ns) $p \geq 0.05$, *$p < 0.05$, **$p < 0.01$, ***$p < 0.001$, ****$p < 0.0001$.

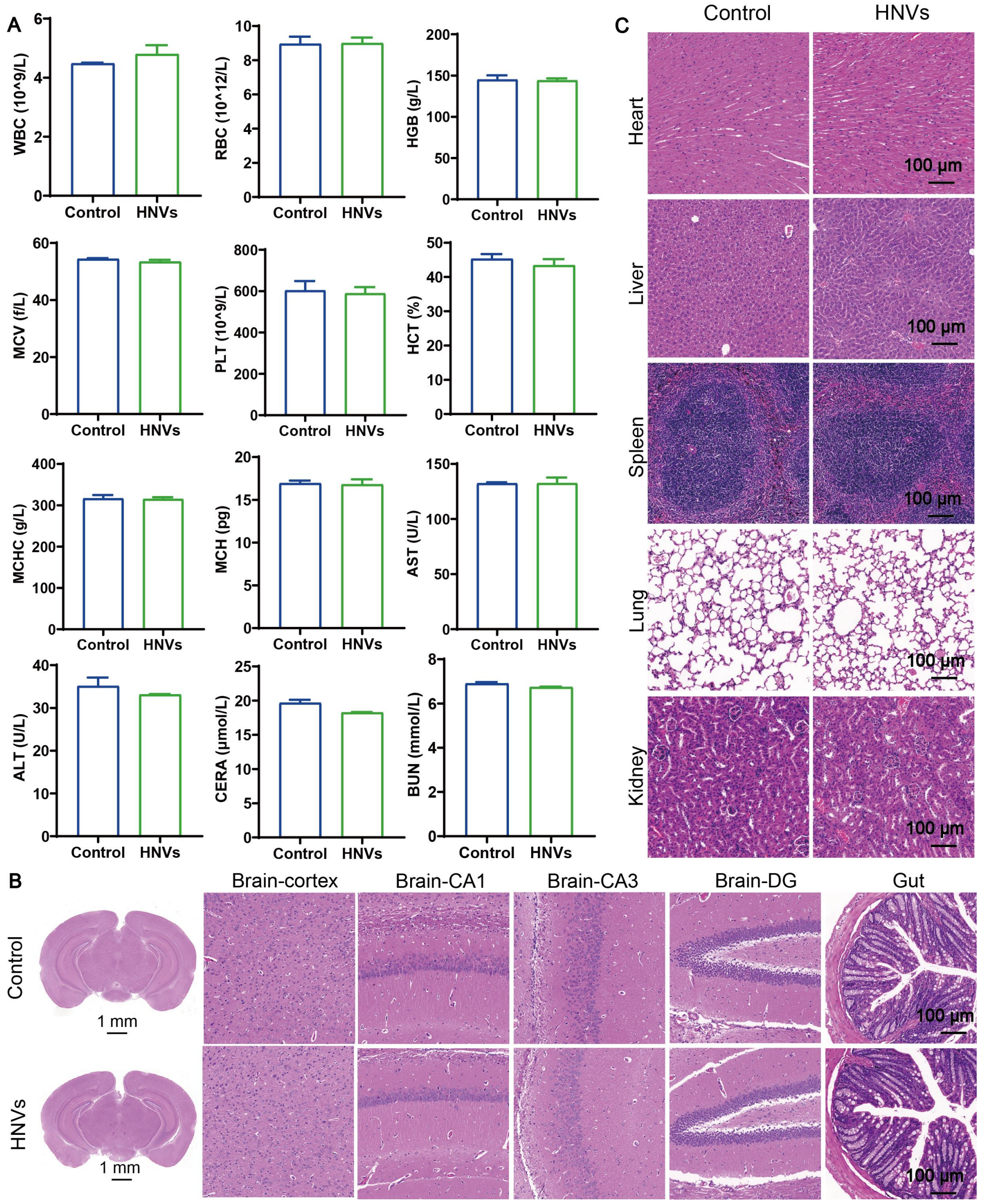

Figure S9. Oral biosafety evaluation of mice after treatment. (A) Hematological and biochemical profiles of mice. WBC, white blood cell count; HGB, hemoglobin; RBC, red blood cell count; MCH, mean corpuscular hemoglobin; MCV, mean cell volume; HCT, hematocrit; MCHC, mean corpuscular hemoglobin concentration; PLT, platelet count; BUN, blood urea nitrogen; AST, aspartate aminotransferase. CREA, creatinine; ALT, alanine aminotransferase (n = 3). (B) H&E staining images of brain tissues from different groups, showing the whole brain (left) and detailed structures of the cerebral cortex and hippocampus (right). Scale bars, 1 mm (left) and 100 μm (right). (C) H&E images of main organs. Scale bars: 100 μm.

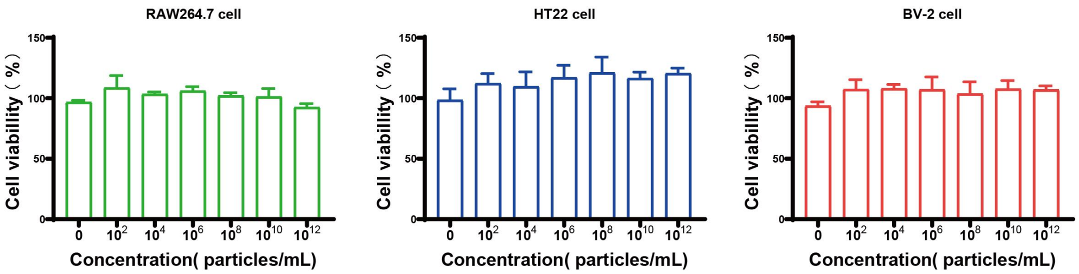

Figure S10. Cell viability assay after treatment with different concentrations of HNVs (n = 3).
